# An innovative strategy for the molecular diagnosis of Usher syndrome identifies causal biallelic mutations in 93% of European patients

**DOI:** 10.1038/ejhg.2016.99

**Published:** 2016-07-27

**Authors:** Crystel Bonnet, Zied Riahi, Sandra Chantot-Bastaraud, Luce Smagghe, Mélanie Letexier, Charles Marcaillou, Gaëlle M Lefèvre, Jean-Pierre Hardelin, Aziz El-Amraoui, Amrit Singh-Estivalet, Saddek Mohand-Saïd, Susanne Kohl, Anne Kurtenbach, Ieva Sliesoraityte, Ditta Zobor, Souad Gherbi, Francesco Testa, Francesca Simonelli, Sandro Banfi, Ana Fakin, Damjan Glavač, Martina Jarc-Vidmar, Andrej Zupan, Saba Battelino, Loreto Martorell Sampol, Maria Antonia Claveria, Jaume Catala Mora, Shzeena Dad, Lisbeth B Møller, Jesus Rodriguez Jorge, Marko Hawlina, Alberto Auricchio, José-Alain Sahel, Sandrine Marlin, Eberhart Zrenner, Isabelle Audo, Christine Petit

**Affiliations:** 1 INSERM UMRS 1120, Institut de la Vision, Paris, France; 2 UPMC-Sorbonnes Universités Paris VI, Paris, France; 3 Service de Génétique et d'Embryologie Médicales, APHP Hôpital Armand Trousseau, Paris, France; 4 INSERM U933, Hôpital Armand Trousseau, Paris, France; 5 IntegraGen SA, Genopole CAMPUS 1 bât. G8, EVRY, Paris, France; 6 Unité de Génétique et Physiologie de l'Audition, Institut Pasteur, Paris, France; 7 INSERM UMRS968, Institut de la Vision, Paris, France; 8 Centre d'Investigation Clinique, Direction de l'Hospitalisation et de l'Organisation des Soins, Centre Hospitalier National d'Ophtalmologie des Quinze-Vingts, Paris, France; 9 Centre for Ophthalmology, Institute for Ophthalmic Research, University of Tuebingen, Tuebingen, Germany; 10 Centre de référence des Surdités Génétiques, Service de Génétique, APHP Hôpital Necker, Paris, France; 11 Eye Clinic, Multidisciplinary Department of Medical, Surgical and Dental Sciences Second University of Naples, Naples, Italy; 12 TIGEM (Telethon Institute of Genetics and Medicine), Pozzuoli, Italy; 13 Department of Biochemistry, Biophysics and General Pathology, Second University of Naples, Naples, Italy; 14 Eye Hospital, University Medical Centre Ljubljana, Ljubljana, Slovenia; 15 Department of Molecular Genetics, Institute of Pathology, University of Ljubljana, Korytkova, Ljubljana; 16 Department of Otorhinolaryngology and Cervicofacial Surgery, University Medical Centre Ljubljana, Zaloska 2, University of Ljubljana, Ljubljana, Slovenia; 17 Hospital Sant Joan de Déu, Barcelona, Spain; 18 Kennedy Center, Gl. Landevej, Glostrup, Denmark; 19 Department of Translational Medicine, "Federico II" University, Napoli, Italy; 20 Werner Reichardt Centre for Integrative Neuroscience (CIN), University of Tuebingen, Tuebingen, Germany; 21 Collège de France, Paris, France

## Abstract

Usher syndrome (USH), the most prevalent cause of hereditary deafness–blindness, is an autosomal recessive and genetically heterogeneous disorder. Three clinical subtypes (USH1–3) are distinguishable based on the severity of the sensorineural hearing impairment, the presence or absence of vestibular dysfunction, and the age of onset of the retinitis pigmentosa. A total of 10 causal genes, 6 for USH1, 3 for USH2, and 1 for USH3, and an USH2 modifier gene, have been identified. A robust molecular diagnosis is required not only to improve genetic counseling, but also to advance gene therapy in USH patients. Here, we present an improved diagnostic strategy that is both cost- and time-effective. It relies on the sequential use of three different techniques to analyze selected genomic regions: targeted exome sequencing, comparative genome hybridization, and quantitative exon amplification. We screened a large cohort of 427 patients (139 USH1, 282 USH2, and six of undefined clinical subtype) from various European medical centers for mutations in all USH genes and the modifier gene. We identified a total of 421 different sequence variants predicted to be pathogenic, about half of which had not been previously reported. Remarkably, we detected large genomic rearrangements, most of which were novel and unique, in 9% of the patients. Thus, our strategy led to the identification of biallelic and monoallelic mutations in 92.7% and 5.8% of the USH patients, respectively. With an overall 98.5% mutation characterization rate, the diagnosis efficiency was substantially improved compared with previously reported methods.

## Introduction

Usher syndrome (USH) is an autosomal recessive disease, accounting for about half of all cases of combined hereditary deafness–blindness. The prevalence of USH has been estimated to be between 1/6000 and 1/25 000.^1, 2^ USH is clinically and genetically heterogeneous. Three clinical subtypes (USH1–3) are distinguishable based on the severity of the hearing impairment, the presence or absence of vestibular dysfunction, and the age of onset of the retinitis pigmentosa associated with the visual deficit.^3^ USH1, the most severe form, results from mutations in any of at least six different genes: *MYO7A* (MIM 276900), *USH1C* (MIM 276904), *CDH23* (MIM 601067)*, PCDH15* (MIM 602083), *USH1G* (MIM 606943), and *CIB2* (MIM 614869), encoding the actin-based motor protein myosin VIIa (USH1B), the transmembrane proteins cadherin-23 (USH1D) and protocadherin-15 (USH1F), the submembrane scaffold proteins harmonin (USH1C) and sans (USH1G), and the calcium-integrin-binding protein CIB2 (USH1J), respectively. There are three known USH2 genes: *USH2A* (MIM 276901), *ADGRV1* (formerly known as *VLGR1* or *GPR98*, MIM 605472), and *DFNB31* (MIM 611383), encoding the large transmembrane proteins usherin (USH2A) and adhesion G-protein-coupled receptor V1 (USH2C), and the submembrane scaffold protein whirlin (USH2D), respectively. In addition, *PDZD7*, encoding a PDZ-domain-containing scaffold protein similar to whirlin and harmonin, is a modifier gene for the retinal phenotype in patients with biallelic mutations in *USH2A*, and contributes to digenic inheritance with *ADGRV1.*^4^ Only one USH3 gene has been identified so far, *CLRN1* (USH3A, MIM 276602), encoding the transmembrane protein clarin-1. Albeit controversial, a fourth clinical subtype has recently been proposed, which regroups atypical forms of USH such as those resulting from mutations of *CEP250*, *HARS*, or *ABHD12*, encoding a centrosomal core protein, an amino acyl tRNA synthetase, and a serine hydrolase, respectively.^5, 6, 7^

USH1 accounts for ~35% of USH cases.^8^ The main five genes implicated in USH1 are *MYO7A* (53–73%), *CDH23* (7–20%), *PCDH15* (7–12%), *USH1C* (1–15%), and *USH1G* (0–4%),^9, 10, 11^ whereas only one USH1J family with a *CIB2* mutation has been identified so far.^12^ USH2, the most frequent USH clinical subtype, accounts for about two-thirds of all reported USH cases.^13^ Mutations in *USH2A*, *ADGRV1*, and *DFNB31* have been identified in 58–90, 5–19, and 0–9.5% of USH2 patients, respectively, depending on the population studied.^9, 10, 14^ Only 3% of USH patients are classified as USH3 in most populations,^15^ except in the Finnish and Ashkenazi Jewish populations: in these populations USH3A accounts for almost 40% of all USH cases.^16, 17^

There are ongoing efforts to find an efficient method for reliable molecular diagnosis of USH. This is essential not only for genetic counseling but also for the development of gene therapy. Genotyping initially focused on a set of single-nucleotide polymorphisms (SNPs) known to be associated with USH, using methods such as arrayed primer extension microarrays. This approach, however, only identified biallelic mutations in about a third of the patients,^18^ which is consistent with the high genetic heterogeneity of USH. To extend the search for sequence variations, we and others then used direct Sanger sequencing to analyze all coding exons and neighboring intronic sequences of USH genes. This allowed the identification of the two causal mutations in ~70% of patients.^9, 10, 14, 19, 20, 21^ However, this approach was time- and cost-consuming because of the large number of exons to be surveyed (~400).^9, 10^ The use of next-generation sequencing techniques, which allow to analyze either the entire exome, or a subset of it, has improved considerably the time- and cost-effectiveness of the molecular diagnosis of USH. The diagnosis efficiency, however, remained ~70% when these techniques were used on their own.^22, 23, 24^ We report here an innovative strategy to identify both short DNA sequence variations and copy number variations (CNVs) using a combinatorial approach. Short DNA sequence variants were identified by high-throughput sequencing of a targeted panel of multiplexed amplicons, and CNVs were identified using genome-wide SNP arrays and exon-specific quantitative real-time polymerase chain reactions (qPCR). This enabled us to substantially improve the efficiency of the molecular diagnosis of USH without significantly increasing the associated time and cost.

## Patients and methods

This study was approved by the local ethics committees, and was carried out following the ethical principles for medical research involving human subjects defined by the WMA Declaration of Helsinki.

### Patients

A total of 427 USH patients were recruited between 2011 and 2015, from six European countries: France (194), Germany (86), Italy (62), Slovenia (61), Spain (21), and Denmark (three). The clinical diagnosis of USH was based on evidence of sensorineural deafness and concurrent retinal degeneration indicative of retinitis pigmentosa. We further classified the patients into USH subtypes based on the results of pure tone audiometry, ocular fundus autofluorescence, and electroretinogram, as well as the presence or absence of a balance defect, manifested in young children by a delay in the ability to sit and walk independently.^25^ All the medical centers shared the same protocol for clinical evaluation of the patients. Patients matching the criteria for the study were enrolled in the cohort once they had agreed (either directly or through their legal representative if underage) to sign an informed consent form and to provide a blood sample for genetic testing. Blood samples from the patient's mother and father were also collected for segregation analysis whenever possible (ie, in ~45% of the cases). Genomic DNA was extracted according to standard procedures. DNA extraction was performed either on site, or after shipment of the blood samples to the research laboratory at the Vision Institute in Paris (France), where all the DNA samples were later processed and analyzed.

### Targeted exome sequencing (TES) and bioinformatic analysis

A multiplex amplicon panel (Fluidigm Access Array) was created to analyze all coding and non-coding exons of the 10 USH genes and the USH2 modifier gene *PDZD7.*^4^ Exons recently identified by retina-specific transcript analysis were included.^26^ The amplicons also covered a minimum of 25-bp intronic sequence flanking each exon to facilitate the detection of sequence variants that affect splice sites. The *USH2A* intronic region harboring the mutation c.7595-2144A>G was also included.^27^ The primers were designed based on the design program Primer3.^28, 29^ A total of 1268 primer pairs (sequences available on request) were chosen to produce amplicons with an average length of 165 bp. Forty-eight pools of primer pairs were created such that each primer pair was represented twice per assay in independent pools, and each pool of primers contained a unique combination of 47–48 different primer pairs.

Following the preparation of the multiplexed amplicon libraries, samples with a minimum of 1 *μ*g of double-stranded DNA, as determined using the SYBR Green I fluorescent double-strand method (Life Technologies, Foster city, CA, USA), were purified using the Agencourt AMPure XP kit (Beckman Coulter Inc., Fullerton, CA, USA). We used an Access Array microfluidic support (Fluidigm, San Francisco, CA, USA) to perform 48 independent PCR reactions in parallel, on 48 different samples at once (ie, a total of 2304 distinct amplicons). We increased the capacity of the device to 110 592 simultaneous PCR reactions per run by optimizing the PCR mix and primer pools to allow multiplexed amplification in each PCR slot. This made it possible to simultaneously produce 2304 amplicons for each of the 48 samples.

During the first PCR on the Access Array, a universal tag present at the 5′ end of each primer (Rd1 Tag on the forward primer and Rd2 Tag on the reverse primer) was added to the extremities of each amplicon. Following thermocycling of the Access Array on the BioMark, the Access Array was transferred to the Post-PCR IFC Controller AX (Fluidigm) to recover the 48 pooled PCRs for each sample. The pooled amplicons were then purified with Agencourt AMPure XP beads and subjected to a second round of PCR, using the universal tags added during the first PCR round as templates. Samples from two distinct Access Arrays were processed at once and subjected to six cycles of amplification in a standard microplate format. This second amplification round was used to add a specific identification barcode to each sample, as well as P5 and P7 adapters for sequencing purposes. Each PCR was then controlled on a Fragment Analyzer (AATI, Ankeny, IA, USA), and quantified to create an equimolar pool of the 96 samples. This pool was again purified with AMPure, and loaded onto a Fragment Analyzer or Bioanalyzer (Agilent, Santa Clara, CA, USA) to verify the profile by comparing it with the expected profile. This pool was sequenced on a HiSeq 2000 sequencer (Illumina, San Diego, CA, USA). For each sample, a total of 313 Mb reads were sequenced per 108 kb of analyzed genome, which represents a 2900 × coverage.

Raw sequencing data were processed for bioinformatics analysis through the Illumina pipeline (CASAVA1.8.2), using the ELANDv2 algorithm for sequence alignment (multiseed and gapped) and the sequence of each amplicon as reference. Variants were called if they met the following criteria: (1) a read depth superior to five with no ambiguous reading, and (2) an allelic frequency inferior to 0.3% in all the following public variant databases: dbSNP132, Hapmap, 1000 Genomes, Exome Variant Server, Exome Aggregation Consortium (http://exac.broadinstitute.org/), Usher-specific Leiden Open Variation Database(https://grenada.lumc.nl/LOVD2/Usher_montpellier/home.php), and Deafness Variation Database (http://deafnessvariationdatabase.org/). They were then ranked according to their expected negative impact on the resulting gene product. Nonsense variants and small deletions or insertions inducing a frameshift of the coding sequence were considered the most damaging, as they necessarily alter the amino-acid sequence of the protein. The pathogenicity of missense and splice-site variants was estimated using the following prediction algorithms: PolyPhen2, SIFT, and Mutation Taster for missence variants, and NNSplice, ESEfinder, Max Ent Scan, Gene Splicer, and Human Splicing Finder for splice-site variants. From those sequence variants predicted to be highly damaging, pathogenic, and/or disease-causing, candidate variants were chosen if they were biallelic and/or lying within genes matching with the clinical diagnosis. Their presence was confirmed in the patient's and, whenever possible, the parents' DNAs, by Sanger sequencing using standard protocols. The entire process, from library preparation to variant identification, took 3–4 weeks for 48 patients.

### Comparative genome hybridization (CGH) using SNP arrays

CytoSNP-12 arrays (Illumina, San Diego, CA, USA), which contain 300 000 polymorphic markers including 200 000 SNPs spread throughout the human genome, were used to assess the zygosity of USH genes in patients, when no mutation or only a monoallelic mutation was detected by TES. The samples were processed using the Infinium assay as previously described^30^ and the results analyzed using Illumina Genome Studio software. An internal reference was created using the clustering algorithm Illumina Gentrain 2.0 on the SNP profiles from 96 samples that were processed during the same run. The SNP profiles were analyzed by comparing the log *R* ratio (where the *R* ratio is the sample copy number over the reference copy number) and B allele frequency (BAF). CNV was identified by the sign of the log *R* ratio. A positive value indicated a duplication and a negative value a deletion. The genotype of each SNP was inferred from the value of its BAF, BAF=0 indicating two copies identical to the reference genome (hg19), whereas BAF=1 signaled two copies of the variant sequence and BAF=0.5, a SNP at the heterozygous state.

### qPCR analysis

Quantitative real-time PCR was performed using a SYBR Green PCR master mix (Applied Biosystems, Foster city, CA, USA) and specific primers were designed using the program Primer3,^28, 29^ such that the amplicon length did not exceed 200 bp (primer sequences available on request). The PCR reactions were performed in duplicate using two different amounts of genomic DNA (10 ng and 2 ng, in 5 *μ*l) in a final volume of 20* μ*l, including 0.2* μ*l of each primer (20 *μ*M final concentration), 4.6 *μ*l of nuclease free water, and 10 μl of power SYBR Green PCR master mix. Positive and negative (no DNA template) controls were used in each run. PCR amplification was performed in a 96-well plate format on a 7500 Fast Real-Time PCR machine (Applied Biosystems) using the following conditions: 2 min at 50 °C, 10 min at 95 °C, 40 cycles (15 s at 95 °C and 1 min at 60 °C), 15 s at 95 °C, 1 min at 60 °C, and 15 s at 95 °C. The final dissociation step was included at the end of the PCR program to generate melting curves and assess primer specificity.^31^ Relative quantification was performed using the ΔΔC_T_ method to normalize the number of USH gene copies to those of the housekeeping gene *GAPDH.*^32, 33^

### Reference sequences for mutation nomenclature and exon numbering

In this article, the nomenclature of all sequence variants and exon numbering refer to the following genomic and cDNA reference sequences [NG_ and NM_ NCBI accession numbers, respectively]: *MYO7A* [NG_009086.1, NM_000260.3] *USH1C* [NG_011883.1, NM_153676.3] *CDH23* [NG_008835.1, NM_022124.5] *PCDH15* [NG_009191.1, NM_001142769.1 (CD2.1 transcript)] *USH1G* [NG_007882.1, NM_173477.4] *CIB2* [NG_033006.1, NM_001301224.1] *USH2A* [NG_009497.1, NM_206933.2] *ADGRV1* [NG_007083.1, NM_032119.1] *WHRN*/*DFNB31* [NG_016700.1, NM_015404.3] *PDZD7* [NG_028030.1, NM_001195263.1] *USH3A* [NG_009168.1, NM_174878.2 (transcript a), NM_052995.2 (transcript c)].

All pathogenic or presumably pathogenic variants identified in the USH genes have been deposited in the ‘Leiden Open Variation Database'.

## Results and discussion

### TES identifies biallelic and monoallelic short sequence variants predicted to be pathogenic in 85% and 12% of USH patients, respectively

TES of the 10 USH genes (6 for USH1, 3 for USH2, 1 for USH3) and the USH2 modifier gene *PDZD7* was carried out. The PCR primers were chosen to allow the multiplex amplification of all known exons of the paneled genes, as well as their flanking intronic sequences (see Patients and Methods for details). A total of 398 exons of interest, representing 108 kb of cumulated genomic sequence, were thus scanned for point mutations and short (<20 bp) deletions or insertions.

First, we validated this TES technique on 12 USH patients previously diagnosed with biallelic point mutations,^10^ and were able to identify all the mutations. We then proceeded to the analysis of a large cohort of 427 clinically defined USH patients, which was established through a collaborative, multicentric initiative involving six European countries: Denmark, France, Germany, Italy, Slovenia, and Spain. For each patient, a list of small sequence variants that were rarely encountered in the general population (allelic frequency inferior to 0.3%) was established (see Patients and Methods for details). This list was then sorted according to (1) the extent of the variant's negative impact on the gene product, (2) the zygosity of the variant, and (3) the location of the variant within a gene consistent with the USH subtype. Variants expected to have a drastic impact on the amino acid sequence because they either introduced a premature stop codon (nonsense variants), or modified the protein sequence, charge, or structure (small frameshift insertions and deletions, splice-site variants, and missense variants), were selected for further evaluation. Then, from this restricted list, the presence of biallelic variants predicted of functional significance was investigated, assessing to this end the zygosity of each variant and the possibility of two different monoallelic variants within the same gene (compound heterozygotes). The adequation of the genetic findings with the initial clinical diagnosis was checked last.

As a result, biallelic mutations were identified in 84.8% (362/427) of the USH patients and monoallelic mutations in an additional 11.7% (50/427) (Table 1 and Supplementary Tables 1–3). Whenever DNA from the proband's parents was available (ie, in ~45% of the cases), the familial segregation analysis confirmed the biallelic inheritance of the mutations (data not shown). Overall, the variants predicted pathogenic were located in every gene surveyed in the panel, except *CIB2* and *DFNB31* (Supplementary Table 4). Notably, 3.5% (15/427) of the patients remained without a detectable mutation at this stage.

### Genome-wide SNP array and gene-focused qPCR analysis identify large genomic rearrangements in 9% of USH patients

When TES failed to identify the biallelic mutations in a patient, the patient's genome was tested for possible large genomic rearrangements by genome-wide SNP array analysis. We first queried allelic imbalance by determining the number of genome copies of 300 000 polymorphic markers distributed throughout the genome. The indication of gains or losses of genetic material in the patient's genome was inferred from the detection of more or fewer SNP copies than expected (see Patients and Methods for details). CNVs covering three or more consecutive SNPs were confirmed by qPCR amplification of the underlying exons. For more ambiguous CNV loci, ie, those identified by only one or two consecutive SNPs and those with unclear boundaries, the amplification of the surrounding exons was also carried out. In parallel, a thorough examination of every exon of the mutation-bearing gene or USH subtype-matching genes of patients with a partial or no molecular diagnosis, respectively, was carried out by qPCR amplification.

**Figure 1:**
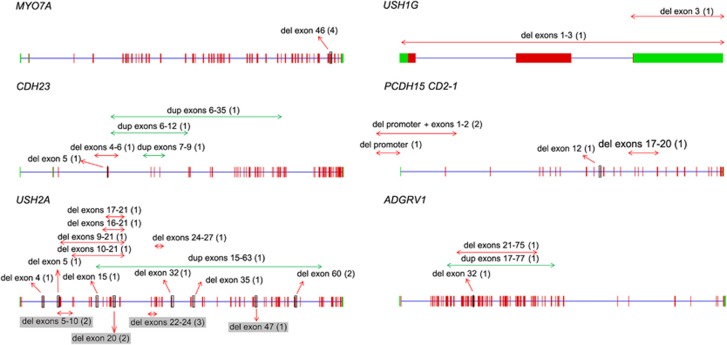
Schematic representation of the large rearrangements identified at USH loci. Novel rearrangements are represented by red (deletion) or green (duplication) left-right arrows, and previously reported rearrangements are highlighted in gray. For each rearrangement, the corresponding number of USH patients from our cohort is indicated between parentheses.

We identified a total of 43 alleles harboring large DNA rearrangements (38 deletions and five duplications), which improved the molecular diagnosis in 60% (39/65) of the USH patients without a complete diagnosis after TES, adding 7.9% of newly resolved cases to the 84.8% of previously resolved cases (Figure 1, Table 1, and Supplementary Table 4). Remarkably, 87% of the large rearrangements identified in this study were novel (Figure 1, red and green annotations), a result largely explained by the systematic survey of the USH gene exons by qPCR whenever genome-wide SNP array analysis was inconclusive. The latter analysis indeed allowed us to identify 21 of the rearrangements (which were all confirmed by qPCR), but the other 22 deletions and insertions were detected only by qPCR analysis of the complete exon sequence of the genes of interest. Of note, only four deletions were biallelic, whereas all the other rearrangements were monoallelic. Segregation analysis was possible for 12 patients and confirmed the biallelic inheritance of the genetic defects (data not shown). Also, 26 deletions and four duplications were found in combination with a monoallelic point mutation. The five remaining monoallelic large rearrangements were not associated with any other detectable mutation.

Therefore, using this three-step strategy to identify both point mutations and genomic rearrangements in USH-associated genes, we identified biallelic mutations in 92.7% (396/427) of the patients (Table 1 and Supplementary Tables 1–2). This corresponds to a raise in the efficiency of the USH molecular diagnosis by 15–25% compared with previous studies.^22, 23, 24^ Regarding the 5.8% (25/427) of the USH patients with as yet incomplete molecular diagnosis (Supplementary Table 3), we assume that we did not detect the second mutation because it lies in a region either not surveyed or not captured by our arrayed PCR techniques. Such regions include introns, promoter regions and other regulatory regions of the TES-surveyed genes, genome-wide areas that are free of CNV markers, and possibly a still unknown USH gene.^22, 34^ In our cohort, only 1.4% (6/427) of the patients characterized clinically as USH1 (one patient) and USH2 (five patients), remain with no molecular diagnosis at the end of the study (data not shown). In addition to the above-mentioned absence or inefficiency of experimental coverage of genome areas, erroneous clinical diagnosis because of an atypical phenotype might also explain the absence of mutations detected in these patients. Finally, we cannot exclude the possibility that some patients suffer from a non-syndromic association of deafness and blindness caused by two unrelated sets of mutations in non-USH genes.

### The proportions of the various types of mutations are different between USH genes

Mutations in USH1 genes were detected in 36% of the USH patients (154/427) (Table 2)*. MYO7A* mutations were detected in 69.5% (107/154), *CDH23* mutations in 13% (20/154), *PCDH15* mutations in 7.8% (12/154), *USH1C* mutations in 7.1% (11/154), and *USH1G* mutations in 2.6% (4/154) of the USH1 patients. We did not detect mutations in *CIB2*. Mutations in USH2 genes were detected in 60.4% of USH patients (258/427) (Table 2). Mutations in *USH2A* and *ADGRV1* were detected in 91.5% (236/258) and 8.1% (21/258) of the USH2 patients, respectively. We did not detect mutations in *DFNB31*.

Of the total 817 allelic variants identified in this study, nonsense or frameshift mutations were detected in 52.1% of the mutated alleles, missense mutations in 31.5%, splice-site mutations in 9.5%, large deletions or insertions in 5.3%, synonymous mutations predicted to result in abnormal splicing in 1%, and in-frame indels and no-stop mutations in 0.6%. However, the proportion of nonsense, frameshift, splice, and missense mutations differed from one USH gene to another (Table 3). This was especially true for the proportion of nonsense mutations, which ranged between 8.1 and 44.5% depending on the USH gene: specifically, 44.5% (4/9) in *USH1G*, 37.5% (15/40) in *ADGRV1*, 31.1% (145/465) in *USH2A*, 26.9% (56/208) in *MYO7A,* 26% (6/23) in *PCDH15*, 19% (4/21) in *USH1C*, 11.7% (2/17) in *CLRN1*, and 8.1% (3/37) in *CDH23*. The proportion of missense mutations was even more variable, ranging from 0–46.6% depending on the USH gene: specifically, 46.6% (97/208) in *MYO7A*, 41.1% (7/17) in *CLRN1*, 37.8% (14/37) in *CDH23*, 28.8% (134/465) in *USH2A*, 10% (4/40) in *ADGRV1*, 4.7% (1/21) in *USH1C*, and 0% in *PCDH15* (0/23) and *USH1G* (0/8). Finally, large DNA rearrangements accounted for 2–33.3% of the mutations depending on the gene: specifically, 33.3% (3/9) in *USH1G,* 26% (6/23) in *PCDH15,* 13.5% (5/37) in *CDH23,* 7.5% (3/40) in *ADGRV1,* 5% (23/465) in *USH2A*, and 2% (4/208) in *MYO7A*. We did not detect any large deletions or duplications in *USH1C*, *CIB2*, *DFNB31*, or *CLRN1*.

**Figure 2:**
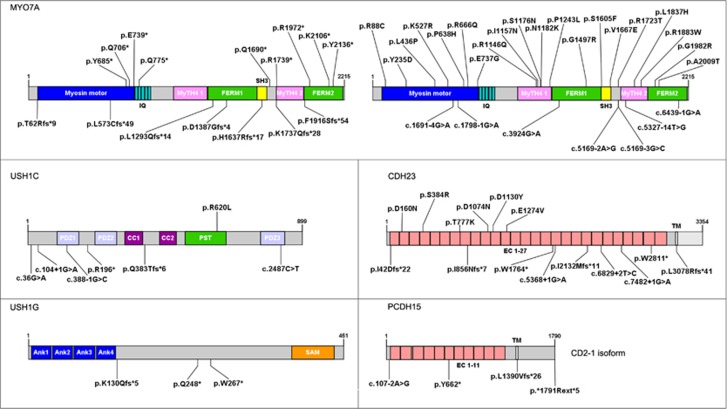
Schematic representation of the proteins encoded by USH1 genes, annotated with the novel variants identified by TES. For each protein, the longest isoform is shown, and the novel pathogenic sequence variants are indicated. Abbreviations: *IQ*, isoleucine-glutamine motifs; *MyTH4*, myosin tail homology 4 domain; *FERM*, band 4.1-ezrin-radixin-moesin domain; *SH3*, src homology 3 domain; *PDZ*, PSD95-discs large-ZO1 domain; *CC*, coiled coil domain; *PST*, proline–serine–threonine–rich region; *EC*, extracellular cadherin domain; *TM*, transmembrane domain; *Ank*, ankyrin domain; *SAM*, sterile alpha motif domain.

**Figure 3:**
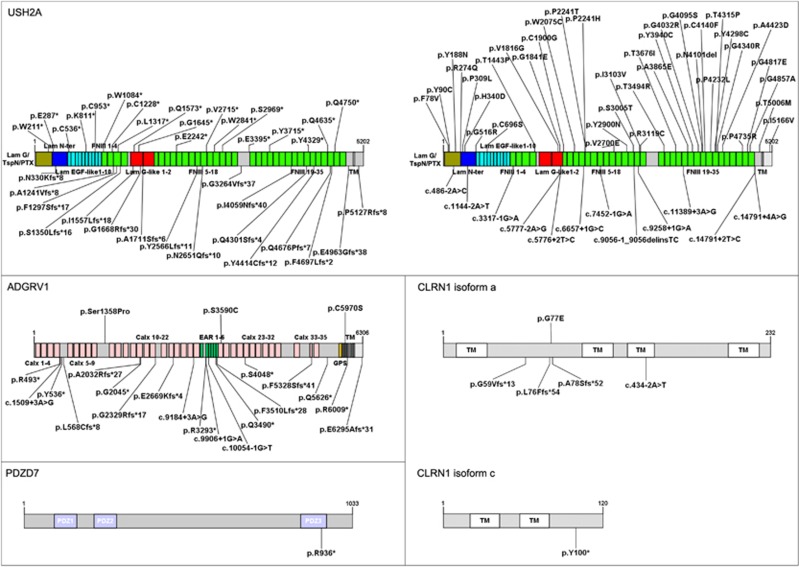
Schematic representation of the proteins encoded by USH2 genes and *PDZD7*, annotated with the novel variants identified by TES. For each protein, the longest isoform is shown, and the novel pathogenic sequence variants are indicated. *LamG/TspN/PTX*, N-terminal thrombospondin/pentaxin/laminin G-like domain; *Lam Nter*, laminin N-terminal domain; *Lam EGF-like*, laminin-type EGF-like domain; *LamG-like*, laminin G-like domain; *FNIII*, fibronectin type III domain; *TM*, transmembrane domain; *Calx*, Ca^2+^-binding calcium exchanger *β*; *EAR*, Epilepsy Associated Repeats; *PDZ*, PSD95-discs large-ZO1 domain; *GPS*, G-protein-coupled proteolysis site.

Remarkably, 213 out of the 421 different mutations identified in this study had not been previously reported (Figures 1, 2, 3, Supplementary Table 4). In our cohort of patients, these new variants represent 40% (*MYO7A*, *USH2A*) to 78% (*USH1G*) of the mutations identified in each USH gene.

### The geographical distribution of recurrent mutations across Europe reveals the existence of regionally restricted mutations

**Figure 4:**
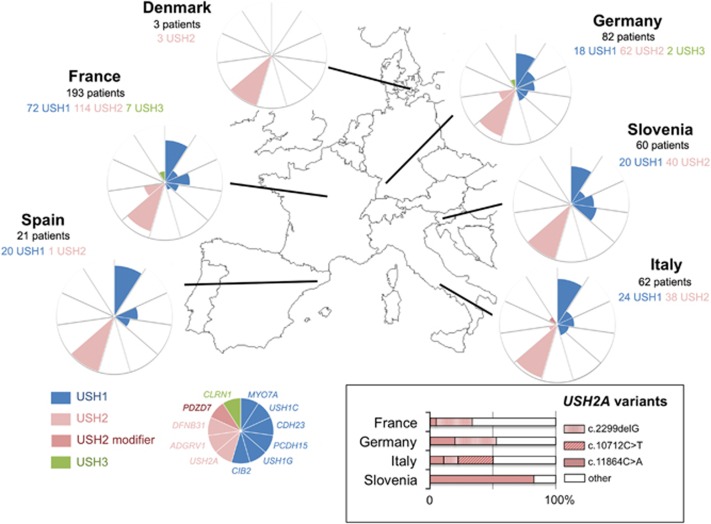
Prevalence and European distribution of the mutations of USH1, USH2, and USH3 genes identified in this study. For each participating country, the pie chart is equally divided in 11 sectors, representing each of the different USH1 (blue), USH2 (pink, and dark pink for the USH2 modifier *PDZD7*), and USH3 (green) genes. In each sector, the colored area indicates the proportion of the USH1 patients, or the proportion of the USH2 and USH3 patients, carrying mutations in the corresponding gene. The inset illustrates the proportion of three prevalent *USH2A* mutations relative to the total number of *USH2A* mutations identified, in France, Germany, Italy, and Slovenia.

Focusing on the mutations present in at least 3% of the patients carrying mutations in a given USH gene, we mostly found recurrent point mutations in *USH2A*, *CDH23*, and *USH1C.* The two most common mutations were *USH2A*:c.2299delG (p.Glu767Serfs*21) and *USH2A*:c.11864G>A (p.Trp3955*). They were detected in ~22% of USH2A patients each (53 and 54 out of 236, respectively), but their distributions differed greatly from one country to another (insert in Figure 4 and Table 4). The proportions of USH2A patients carrying the c.2299delG variant and the c.11864G>A variant were 32.7% (18/55) and 20% (11/55) in Germany, 28.7% (29/101) and 4.9% (5/101) in France, 11.1% (4/36) and 11.1% (4/36) in Italy, and 0% (0/40) and 82.5% (33/40) in Slovenia, respectively. Other recurrent mutations were detected in *USH2A*, *CDH23*, and *USH1C* throughout Europe, and are presented in Table 4. Of note, five recurrent point mutations were geographically restricted: *MYO7A*:c.721C>G (p.Arg241Gly) and *USH2A*:c.10712C>T (p.Thr3571Met) to Italy, *MYO7A*:c.52C>T (p.Gln18*) and *PCDH15*:c.1103delT (p.Leu368Trpfs*58) to Slovenia, and *USH2A*:c.2276G>T (p.Cys759Phe) to France (3.9% of USH2A patients) (Table 4). The variant *MYO7A*:c.721C>G (p.Arg241Gly) had already been reported in Italian patients^35^ and the variants *USH2A*:c.10712C>T (p.Thr3571Met) and *USH2A*:c.2276G>T (p.Cys759Phe) had been reported in other European populations.^21, 36^ The variant *MYO7A*:c.52C>T (p.Gln18*) had been previously reported in Japan and Canada,^37, 38^ but never before in Europe. Finally, the variant *PCDH15*:c.1103delT (p.Leu368Trpfs*58), identified exclusively in Slovenian patients in this study, had only been found before in the Hutterite population originating from Moravia.^39^

We also identified possibly recurrent large deletions on the basis of exon loss. We detected a deletion in *MYO7A,* which encompasses exon 46, in four patients from three different countries. We also detected three different deletions in *USH2A,* encompassing exons 22–24, exon 20, or exon 60, and a deletion in *PCDH15*, encompassing its promoter region and first coding exon, in two unrelated patients each. The characterization of the precise breakpoints of these deletions will clarify whether they are related to the existence of abnormal recombination hot spots.

### The presence, in a few patients, of mutations in more than one USH gene calls for thorough molecular diagnosis

We noticed that four patients carried, on top of biallelic mutations in a given USH gene, an additional deleterious monoallelic variant in a different USH gene. One patient was homozygous for *USH2A*:c.1876C>T (p.Arg626*) and heterozygous for *USH1G*:c.800G>A (p.Trp267*). Another patient was a compound heterozygote for *MYO7A*:c.3503G>A (p.Arg1168Gln)+deletion of exon 46, and was heterozygous for *USH2A*:c.2299delG (p.Glu767Serfs*21). The third patient, who was a compound heterozygote *USH2A*:c.9014G>C (p.Ser3005Thr)+deletion of exon 4, also carried a monoallelic large duplication encompassing exon 6–37 of *CDH23*. Finally, the last patient was homozygous for *MYO7A*:c.2476G>A (p.Ala826Thr) and heterozygous for *ADGRV1*:c.10768A>T (p.Ser3590Cys). This points to the need to screen all USH and USH-associated genes to avoid rendering an inaccurate molecular diagnosis, which could compromise genetic counseling and possibly also gene therapy. However, we did not find compelling genetic evidence for a digenic transmission of the USH phenotype in any patient of this cohort.

*PDZD7* is a modifier gene of the retinal phenotype in USH2A patients, and contributes to the digenic inheritance of USH2 with *ADGRV1.*^4^ We did not detect any sequence variants of functional significance in *PDZD7* in any of the 236 USH2A patients or of the three USH2C patients with a monoallelic mutation of *ADGRV1*. However, we did identify the *PDZD7*:c.2806C>T (p.Arg936*) variant at the heterozygous state in an Italian patient with a clinical diagnosis of USH2, but this patient did not carry any other sequence variant of functional significance in the USH genes analyzed.

### Patients clinically diagnosed as USH2 may carry mutations in USH1 genes

Our cohort consisted of 139 and 282 patients classified as USH1 and USH2 on clinical criteria, respectively, and six unclassified patients. On the other side, the molecular analysis gave the following distribution of biallelic or monoallelic mutations in USH genes: 154 USH1, 258 USH2, and 9 USH3. This evidenced a genotype-phenotype discrepancy in 13 patients initially classified as USH2 (marked with a # next to their ID code in Supplementary Tables 1 and 3), who turned out to carry biallelic mutations in USH1 genes: specifically, *CDH23* (seven patients), *MYO7A* (five patients), and *USH1C* (one patient). This indicates that mutations in these genes can lead to phenotypes that cross the boundaries between the USH clinical subtypes. In fact, there does not appear to be a straightforward correlation between the severity of the USH phenotype, especially that of the hearing impairment, and the type of mutations identified in these genes. In addition, eight of the nine patients carrying mutations in the USH3 gene had been classified as USH2 on clinical criteria, and the last one was unclassified. Incidentally, the remaining five patients initially unclassified carried mutations in USH1 genes (three patients) or USH2 genes (two patients).

### Comparison of this strategy with previous strategies for the molecular diagnosis of USH

Technical improvements for the molecular diagnosis of USH have been recently reported. Besnard *et al.*^23^ used massively parallel targeted sequencing and found biallelic mutations in 77% (10/13) of European patients. Bujakowska *et al.*^40^ performed selective exon capture, followed by NGS and CGH array analysis, but surprisingly, detected biallelic mutations in barely a third (29.7%) of the 47 USH1 patients tested. Krawitz *et al.*^22^ used targeted enrichment and deep sequencing of USH exons and identified biallelic mutations in 79% (35/44) of the patients. Likewise, Aparisi *et al.*^24^ identified biallelic mutations in 68% (22/32) of USH patients. The strategy described here allowed us to raise the overall diagnostic effectiveness by 15–25%, compared with these studies. Arrayed PCR-based TES provided an excellent coverage of the exons analyzed, and the use of qPCR to detect large genomic rearrangements that could not be identified by TES was very efficient. Indeed, using qPCR, we not only confirmed 21 deletions or insertions found by SNP array analysis, but also uncovered 23 additional rearrangements that were overlooked by the genome-wide SNP array analysis. Previous studies had led to the identification of large deletions or duplications in *USH2A* and *PCDH15*, but always on relatively small cohorts of USH patients.^22, 41, 42, 43^ The strategy used in this study allowed us to identify large deletions or duplications not only in *USH2A* and *PCDH15,* but also in *CDH23*, *MYO7A*, *ADGRV1*, and *USH1G* (Figure 1), bringing the total to 43 rearrangements present in 9.1% (39 out of 427) of the patients studied.

In conclusion, the combined use of two distinct approaches aiming at the identification, on the one hand, of small nucleotide variations, using an arrayed PCR-based technique and high-throughput DNA sequencing, and on the other hand, of large genomic rearrangements, using the SNP array technique coupled with exon-specific qPCR analysis, substantially improved the quality of the molecular diagnosis of USH, resulting in the highest diagnostic yield ever obtained, on the largest cohort of USH patients studied to date.

## Figures and Tables

**Table 1:**
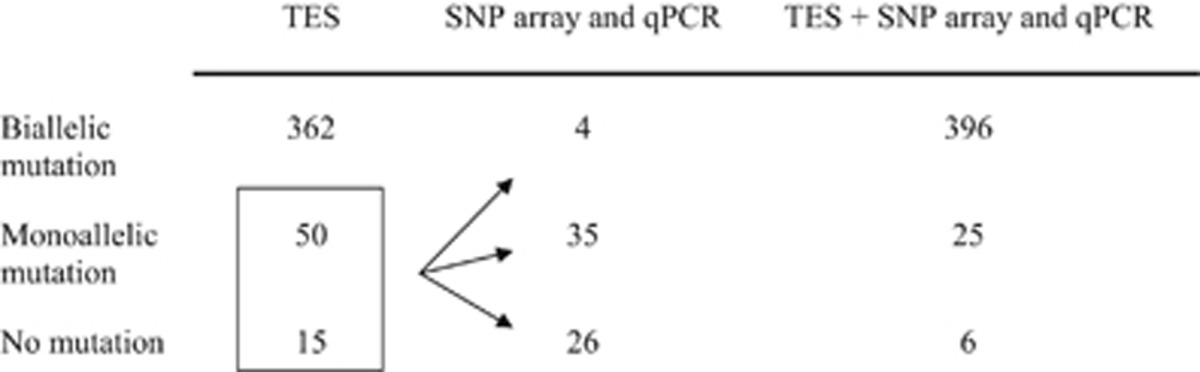
Number of patients with biallelic, monoallelic, and no mutations of functional significance in USH genes identified by the three techniques (TES, SNP array, and qPCR) used sequentially

**Table 2:** Distribution of the USH patients according to their clinical subtype and, for each subtype, the affected gene

*Clinical subtype*	*Gene*	*Number of patients*
USH1 (36%)	*MYO7A*	107 (69.5%)
	*CDH23*	20 (13%)
	*PCDH15*	12 (7.8%)
	*USH1C*	11 (7.1%)
	*USH1G*	4 (2.6%)
USH2 (60.4%)	*USH2A*	236 (91.5%)
	*ADGRV1*	21 (8.1%)
	*PDZD7*	1 (0.4%)
USH3 (2.1%)	*CLRN1*	9

**Table 3:**
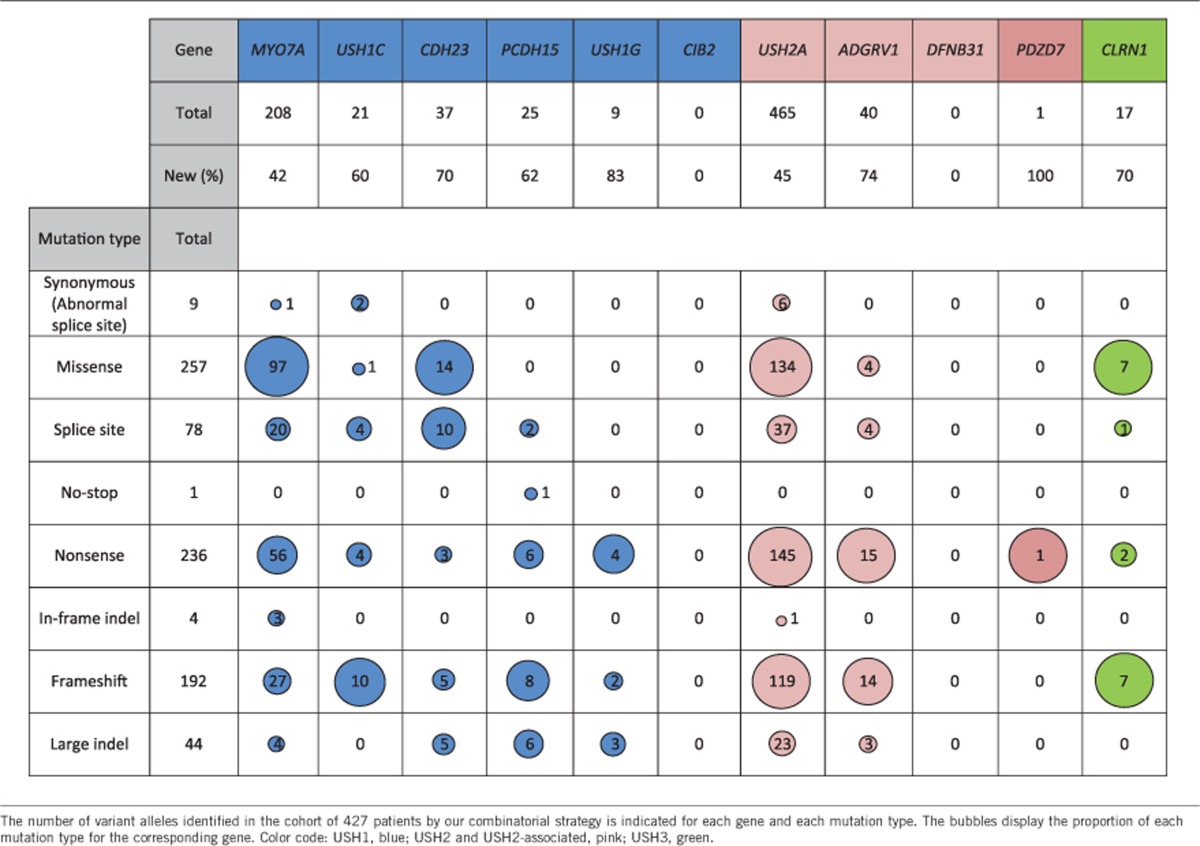
Number of variant alleles identified for each USH gene, and for the different mutation types

**Table 4:**
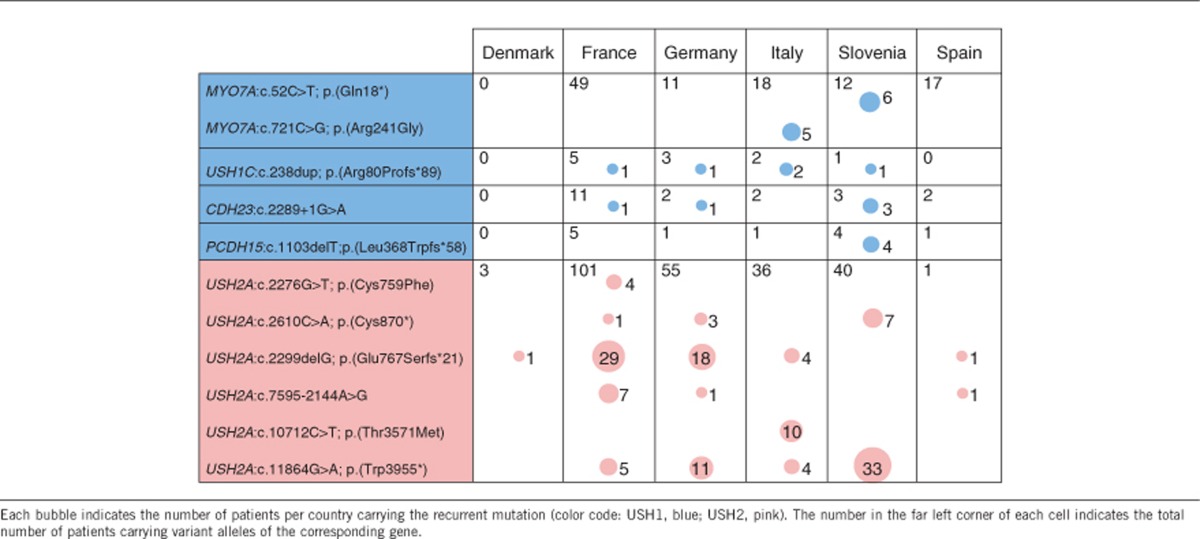
Recurrent mutations in USH1 genes and *USH2A*

## References

[bib1] Boughman JA, Vernon M, Shaver KA: Usher syndrome: definition and estimate of prevalence from two high-risk populations. J Chronic Dis 1983; 36: 595–603. 6885960 10.1016/0021-9681(83)90147-9

[bib2] Kimberling WJ, Hildebrand MS, Shearer AE et al: Frequency of Usher syndrome in two pediatric populations: Implications for genetic screening of deaf and hard of hearing children. Genet Med 2010; 12: 512–516. 20613545 10.1097/GIM.0b013e3181e5afb8 PMC3131500

[bib3] Bonnet C, El-Amraoui A: Usher syndrome (sensorineural deafness and retinitis pigmentosa): pathogenesis, molecular diagnosis and therapeutic approaches. Curr Opin Neurol 2012; 25: 42–49. 22185901 10.1097/WCO.0b013e32834ef8b2

[bib4] Ebermann I, Phillips JB, Liebau MC et al: *PDZD7* is a modifier of retinal disease and a contributor to digenic Usher syndrome. J Clin Investig 2010; 120: 1812–1823. 20440071 10.1172/JCI39715 PMC2877930

[bib5] Khateb S, Zelinger L, Mizrahi-Meissonnier L et al: A homozygous nonsense *CEP250* mutation combined with a heterozygous nonsense *C2orf71* mutation is associated with atypical Usher syndrome. J Med Genet 2014; 51: 460–469. 24780881 10.1136/jmedgenet-2014-102287

[bib6] Nishiguchi KM, Avila-Fernandez A, van Huet RA et al: Exome sequencing extends the phenotypic spectrum for *ABHD12* mutations: from syndromic to nonsyndromic retinal degeneration. Ophthalmology 2014; 121: 1620–1627. 24697911 10.1016/j.ophtha.2014.02.008

[bib7] Puffenberger EG, Jinks RN, Sougnez C et al: Genetic mapping and exome sequencing identify variants associated with five novel diseases. PloS One 2012; 7: e28936. 22279524 10.1371/journal.pone.0028936 PMC3260153

[bib8] Petit C: Usher syndrome: from genetics to pathogenesis. Ann Rev Genomics Hum Genet 2001; 2: 271–297. 11701652 10.1146/annurev.genom.2.1.271

[bib9] Le Quesne Stabej P, Saihan Z, Rangesh N et al: Comprehensive sequence analysis of nine Usher syndrome genes in the UK National Collaborative Usher Study. J Med Genet 2012; 49: 27–36. 22135276 10.1136/jmedgenet-2011-100468 PMC3678402

[bib10] Bonnet C, Grati M, Marlin S et al: Complete exon sequencing of all known Usher syndrome genes greatly improves molecular diagnosis. Orphanet J Rare Dis 2011; 6: 21. 21569298 10.1186/1750-1172-6-21 PMC3125325

[bib11] Roux AF, Faugere V, Vache C et al: Four-year follow-up of diagnostic service in USH1 patients. Invest Ophthalmol Vis Sci 2011; 52: 4063–4071. 21436283 10.1167/iovs.10-6869

[bib12] Riazuddin S, Belyantseva IA, Giese AP et al: Alterations of the CIB2 calcium- and integrin-binding protein cause Usher syndrome type 1J and nonsyndromic deafness DFNB48. Nat Genet 2012; 44: 1265–1271. 23023331 10.1038/ng.2426 PMC3501259

[bib13] Eudy JD, Yao S, Weston MD et al: Isolation of a gene encoding a novel member of the nuclear receptor superfamily from the critical region of Usher syndrome type IIa at 1q41. Genomics 1998; 50: 382–384. 9676434 10.1006/geno.1998.5345

[bib14] Garcia-Garcia G, Besnard T, Baux D et al: The contribution of *GPR98* and *DFNB31* genes to a Spanish Usher syndrome type 2 cohort. Mol Vis 2013; 19: 367–373. 23441107 PMC3580968

[bib15] Millan JM, Aller E, Jaijo T, Blanco-Kelly F, Gimenez-Pardo A, Ayuso C: An update on the genetics of Usher syndrome. J Ophthalmol 2011; 2011: 417217. 21234346 10.1155/2011/417217 PMC3017948

[bib16] Ness SL, Ben-Yosef T, Bar-Lev A et al: Genetic homogeneity and phenotypic variability among Ashkenazi Jews with Usher syndrome type III. J Med Genet 2003; 40: 767–772. 14569126 10.1136/jmg.40.10.767 PMC1735287

[bib17] Joensuu T, Hamalainen R, Yuan B et al: Mutations in a novel gene with transmembrane domains underlie Usher syndrome type 3. Am J Hum Genet 2001; 69: 673–684. 11524702 10.1086/323610 PMC1226054

[bib18] Jaijo T, Aller E, Garcia-Garcia G et al: Microarray-based mutation analysis of 183 Spanish families with Usher syndrome. Invest Ophthalmol Vis Sci 2010; 51: 1311–1317. 19683999 10.1167/iovs.09-4085

[bib19] Roux AF, Faugere V, Le Guedard S et al: Survey of the frequency of USH1 gene mutations in a cohort of Usher patients shows the importance of cadherin 23 and protocadherin 15 genes and establishes a detection rate of above 90%. J Med Genet 2006; 43: 763–768. 16679490 10.1136/jmg.2006.041954 PMC2564578

[bib20] Dreyer B, Brox V, Tranebjaerg L et al: Spectrum of *USH2A* mutations in Scandinavian patients with Usher syndrome type II. Hum Mutat 2008; 29: 451. 10.1002/humu.9524 18273898

[bib21] Aller E, Jaijo T, Beneyto M et al: Identification of 14 novel mutations in the long isoform of USH2A in Spanish patients with Usher syndrome type II. J Med Genet 2006; 43: e55. 17085681 10.1136/jmg.2006.041764 PMC2563181

[bib22] Krawitz PM, Schiska D, Kruger U et al: Screening for single nucleotide variants, small indels and exon deletions with a next-generation sequencing based gene panel approach for Usher syndrome. Mol Genet Genomic Med 2014; 2: 393–401. 25333064 10.1002/mgg3.92 PMC4190874

[bib23] Besnard T, Garcia-Garcia G, Baux D et al: Experience of targeted Usher exome sequencing as a clinical test. Mol Genet Genomic Med 2014; 2: 30–43. 24498627 10.1002/mgg3.25 PMC3907913

[bib24] Aparisi MJ, Aller E, Fuster-Garcia C et al: Targeted next generation sequencing for molecular diagnosis of Usher syndrome. Orphanet J Rare Dis 2014; 9: 168. 25404053 10.1186/s13023-014-0168-7 PMC4245769

[bib25] Smith RJ, Berlin CI, Hejtmancik JF et al: Clinical diagnosis of the Usher syndromes. Usher Syndrome Consortium. Am J Med Genet 1994; 50: 32–38. 8160750 10.1002/ajmg.1320500107

[bib26] Farkas MH, Grant GR, White JA, Sousa ME, Consugar MB, Pierce EA: Transcriptome analyses of the human retina identify unprecedented transcript diversity and 3.5 Mb of novel transcribed sequence via significant alternative splicing and novel genes. BMC Genomics 2013; 14: 486. 23865674 10.1186/1471-2164-14-486 PMC3924432

[bib27] Vache C, Besnard T, le Berre P et al: Usher syndrome type 2 caused by activation of an *USH2A* pseudoexon: implications for diagnosis and therapy. Hum Mutat 2012; 33: 104–108. 22009552 10.1002/humu.21634

[bib28] Untergasser A, Cutcutache I, Koressaar T et al: Primer3: new capabilities and interfaces. Nucleic Acids Res 2012; 40: e115. 22730293 10.1093/nar/gks596 PMC3424584

[bib29] Koressaar T, Remm M: Enhancements and modifications of primer design program Primer3. Bioinformatics 2007; 23: 1289–1291. 17379693 10.1093/bioinformatics/btm091

[bib30] Steemers FJ, Chang W, Lee G, Barker DL, Shen R, Gunderson KL: Whole-genome genotyping with the single-base extension assay. Nat Methods 2006; 3: 31–33. 16369550 10.1038/nmeth842

[bib31] D'Haene B, Vandesompele J, Hellemans J: Accurate and objective copy number profiling using real-time quantitative PCR. Methods 2010; 50: 262–270. 20060046 10.1016/j.ymeth.2009.12.007

[bib32] Livak KJ, Schmittgen TD: Analysis of relative gene expression data using real-time quantitative PCR and the 2^−ΔΔ*C*^_T_ method. Methods 2001; 25: 402–408. 11846609 10.1006/meth.2001.1262

[bib33] Schefe JH, Lehmann KE, Buschmann IR, Unger T, Funke-Kaiser H: Quantitative real-time RT-PCR data analysis: current concepts and the novel "gene expression's *C*_T_ difference" formula. J Mol Med 2006; 84: 901–910. 16972087 10.1007/s00109-006-0097-6

[bib34] Ahmed ZM, Riazuddin S, Bernstein SL et al: Mutations of the protocadherin gene *PCDH15* cause Usher syndrome type 1F. Am J Hum Genet 2001; 69: 25–34. 11398101 10.1086/321277 PMC1226045

[bib35] Jaijo T, Aller E, Beneyto M et al: MYO7A mutation screening in Usher syndrome type I patients from diverse origins. J Med Genet 2007; 44: e71. 17361009 10.1136/jmg.2006.045377 PMC2598023

[bib36] Dreyer B, Tranebjaerg L, Rosenberg T, Weston MD, Kimberling WJ, Nilssen O: Identification of novel *USH2A* mutations: implications for the structure of USH2A protein. Eur J Hum Genet 2000; 8: 500–506. 10909849 10.1038/sj.ejhg.5200491

[bib37] Zhou Q, Lenger C, Smith R et al: Evidence of genetic heterogeneity in Alberta Hutterites with Usher syndrome type I. Mol Vis 2012; 18: 1379–1383. 22690115 PMC3369897

[bib38] Yoshimura H, Iwasaki S, Kanda Y et al: An Usher syndrome type 1 patient diagnosed before the appearance of visual symptoms by *MYO7A* mutation analysis. Int J Pediatr Otorhinolaryngol 2013; 77: 298–302. 23237960 10.1016/j.ijporl.2012.11.007

[bib39] Alagramam KN, Yuan H, Kuehn MH et al: Mutations in the novel protocadherin PCDH15 cause Usher syndrome type 1F. Hum Mol Genet 2001; 10: 1709–1718. 11487575 10.1093/hmg/10.16.1709

[bib40] Bujakowska KM, Consugar M, Place E et al: Targeted exon sequencing in Usher syndrome type I. Invest Ophthalmol Vis Sci 2014; 55: 8488–8496. 25468891 10.1167/iovs.14-15169 PMC4280089

[bib41] Steele-Stallard HB, Le Quesne Stabej P, Lenassi E et al: Screening for duplications, deletions and a common intronic mutation detects 35% of second mutations in patients with *USH2A* monoallelic mutations on Sanger sequencing. Orphanet J Rare Dis 2013; 8: 122. 23924366 10.1186/1750-1172-8-122 PMC3751126

[bib42] Shzeena D, Rendtorff ND, Kann E et al: Partial *USH2A* deletions contribute to Usher syndrome in Denmark. Eur J Hum Genet 2015; 23: 1750. 10.1038/ejhg.2015.131 PMC4795201 26559128

[bib43] Le Guedard S, Faugere V, Malcolm S, Claustres M, Roux AF: Large genomic rearrangements within the *PCDH15* gene are a significant cause of USH1F syndrome. Mol Vis 2007; 13: 102–107. 17277737 PMC2533038

