# Supplementary material for: An innovative strategy for the molecular diagnosis of Usher syndrome identifies causal biallelic mutations in 93% of European patients
Source: Eur J Hum Genet. 2016 Jul 27;24(12):1730–8. doi: 10.1038/ejhg.2016.99 (PMC5117943; doi:10.1038/ejhg.2016.99)
Supplement: Supplementary Table 1 [file ejhg201699x1.docx]

**Supplementary Table 1: Genotype of the USH1 patients**

| **Patient Number** | **Gene** | **Allele 1** | **Allele 2** | | | **Additional mutation** |
| --- | --- | --- | --- | --- | --- | --- |
|  |  |  |  | |  | |
| FR02C016402 | *CDH23* | **p.(Leu3078Argfs*41)** | c.6050-9G>A | |  | |
| FR02C0310400^#^ | *CDH23* | **p.(Ser384Arg)** | **p.(Ser384Arg)** | |  | |
| FR02C0429592 | *CDH23* | p.(Gly2017Ser) | p.(Gly2017Ser) | |  | |
| FR02C0513052320 | *CDH23* | **p.(Glu1274Val)** | **p.(Glu1274Val)** | |  | |
| FR02C06S47211 | *CDH23* | p.(Gln1123*) | p.(Thr1587Cysfs*4) | |  | |
| FR02C07U2260^#^ | *CDH23* | **p.(Trp1764*)** | **p.(Asp1074Asn)** | |  | |
| FR02C07U2420 | *CDH23* | **p.(Asp160Asn)** | **p.(Asp160Asn)** | |  | |
| FR02C02U2451 | *CDH23* | c.2289+1G>A | c.6050-9G>A | |  | |
| DE03C02591 | *CDH23* | **c.5368+1G>A** | **c.(145+1_146-1)_(429+1_430-1)del** | |  | |
| IT02C0113700^#^ | *CDH23* | **p.(Thr777Lys)** | p.(His755Tyr) | |  | |
| IT02C021860 | *CDH23* | **p.(Asp1130Tyr)** | **p.(Asp1130Tyr)** | |  | |
| SLO01C0111232^#^ | *CDH23* | c.2289+1G>A | **c.(429+1_430-1)_(832+1_833-1)dup** | |  | |
| SLO01C0212152^#^ | *CDH23* | **p.(Ile856Asnfs*7)** | c.2289+1G>A | |  | |
| SLO01C0312522 | *CDH23* | c.2289+1G>A | **c.(288+1_289-1)_(336+1_337-1)del** | |  | |
| SP01C011515260 | *CDH23* | **p.(Ile42Aspfs*22)** | **c.6829+2T>C** | |  | |
| SP01C025111011 | *CDH23* | **p.(Trp2811*)** | p.(Asp2148Asn) | |  | |
| FR02M01S57520 | *MYO7A* | **p.(Arg1723Thr)** | **p.(Arg1723Thr)** | |  | |
| FR02M02A13260 | *MYO7A* | **c.4323+2T>G** | p.(Gln1798*) | |  | |
| FR02M04A19080 | *MYO7A* | p.(Leu2186Pro) | p.(Leu2186Pro) | |  | |
| FR02M073092 | *MYO7A* | **p.(Leu436Pro)** | p.(Gly214Arg) | |  | |
| FR02M094172 | *MYO7A* | p.(Gly163Arg) | p.(Ala198Thr) | |  | |
| FR02M1027292 | *MYO7A* | p.(Ala826Thr ) | p.(Ala826Thr ) | | *ADGRV1* **p.(Ser3590Cys)** | |
| FR02M1130792 | *MYO7A* | p.(Arg1240Gln) | p.(Arg1240Gln) | |  | |
| FR02M0532222 | *MYO7A* | p.(Leu2186Pro) | p.(Leu2186Pro) | |  | |
| FR02M1636722 | *MYO7A* | c.2283-1G>T | c.2283-1G>T | |  | |
| FR02M1941792 | *MYO7A* | **p.(Ile1157Asn)** | **p.(Ile1157Asn)** | |  | |
| FR02M2042902 | *MYO7A* | **p.(Leu1293Glnfs*14)** | p.(Gly955Ser ) | |  | |
| FR02M2148372 | *MYO7A* | **p.(Gly1982Arg)** | **p.(Gly1982Arg)** | |  | |
| FR02M2255032 | *MYO7A* | **p.(Thr62Argfs*9)** | p.(Lys1255Argfs*8) | |  | |
| FR02M2356442 | *MYO7A* | **c.6439-1G>A** | **c.6439-1G>A** | |  | |
| FR02M2464712 | *MYO7A* | p.(Arg1168Gln) | **c.(6237+1_6238-1)_(6354+1_6355-1)del** | *USH2A* p.(Glu767Serfs*21) | | |
| FR02M1765182 | *MYO7A* | c.2283-1G>T | c.2283-1G>T | |  | |
| FR02M1565292 | *MYO7A* | p.(Arg150*) | **p.(His1637Argfs*17)** | |  | |
| FR02M2667312 | *MYO7A* | **p.(Trp1972*)** | p.(Arg241His) | |  | |
| FR02M2768332 | *MYO7A* | **p.(Lys527Arg)** | **p.(Pro1243Leu)** | |  | |
| FR02M2879862 | *MYO7A* | **p.(Arg666Gln)** | **p.(Ser1176Asn)** | |  | |
| FR02M2983562 | *MYO7A* | p.(Lys420*) | p.(Lys420*) | |  | |
| FR02M3013051831 | *MYO7A* | **c.5169-3G>C** | p.(Gly1942Arg) | |  | |
| FR02M3114012200 | *MYO7A* | p.(Arg1967*) | **c.5327-14T>G** | |  | |
| FR02M3214026820 | *MYO7A* | p.(Arg212His) | p.(Arg212His) | |  | |
| FR02M3414030010 | *MYO7A* | **p.(Leu1837His)** | **p.(Leu1837His)** | |  | |
| FR02M3514031581 | *MYO7A* | p.(Arg972*) | p.(Arg972*) | |  | |
| FR02M0314035231 | *MYO7A* | p.(Gln1798*) | p.(Phe1946Serfs*23) | |  | |
| FR02M0814048991 | *MYO7A* | c.133-2A>G | p.(Gly214Arg) | |  | |
| FR02M1815002630 | *MYO7A* | p.(Cys1198*) | p.(Lys269del) | | **p.(Phe1346del)** | |
| FR02M36S38511 | *MYO7A* | **p.(Val1667Glu)** | **p.(Val1667Glu)** | |  | |
| FR02M37S51890 | *MYO7A* | **p.(Phe1916Serfs*54)** | **p.(Phe1916Serfs*54)** | |  | |
| FR02M38S65600 | *MYO7A* | p.(Arg1873Trp) | p.(Arg1873Trp) | |  | |
| FR02M39S8521 | *MYO7A* | **p.(Asp1387Glyfs*4)** | **p.(Asp1387Glyfs*4)** | |  | |
| FR02M40U1050 | *MYO7A* | **p.(Leu573Cysfs*49)** | **p.(Leu573Cysfs*49)** | |  | |
| FR02M41U1170 | *MYO7A* | p.(Ala2009Profs*32) | c.1690+1G>A | |  | |
| FR02M42U1451 | *MYO7A* | **p.(Arg1739*)** | **p.(Arg1739*)** | |  | |
| FR02M43U1741 | *MYO7A* | p.(Asp2010Asn) | p.(Asp2010Asn) | |  | |
| FR02M45U1771^#^ | *MYO7A* | p.(Arg669*) | **p.(Ala2009Thr)** | |  | |
| FR02M33U1801 | *MYO7A* | p.(Tyr333*) | p.(His133Profs*7) | |  | |
| FR02M44U1881 | *MYO7A* | p.(Gly25Arg ) | p.(Asp2010Asn) | |  | |
| FR02M46U2041 | *MYO7A* | **p.(Arg88Cys)** | **p.(Lys2106*)** | |  | |
| FR02M12U2270 | *MYO7A* | p.(Phe1963del) | p.(Arg1240Gln) | |  | |
| FR02M47U2320 | *MYO7A* | c.2695-9A>G | c.2695-9A>G | |  | |
| FR02M13U2381 | *MYO7A* | p.(Arg1240Gln) | p.(Arg150*) | |  | |
| FR02M48U2480 | *MYO7A* | p.(Gln234*) | p.(Gln234*) | |  | |
| FR02M25U2620 | *MYO7A* | **p.(Arg1146Gln)** | **c.(6237+1_6238-1)_(6354+1_6355-1)del** | |  | |
| FR02M49U680 | *MYO7A* | **p.(Asn1182lys)** | **p.(Asn1182lys)** | |  | |
| FR02M06U911 | *MYO7A* | p.(Leu2186Pro) | p.(Thr165Met) | |  | |
| DE03M091051^#^ | *MYO7A* | **c.5169-2A>G** | **c.1798-1G>A** | |  | |
| DE03M01110 | *MYO7A* | p.(Thr165Met) | **p.(Glu737Gly)** | |  | |
| DE03M041322 | *MYO7A* | **p.(Tyr685*)** | p.(Arg1240Gln) | |  | |
| DE03M08180 | *MYO7A* | p.(Arg1873Trp) | p.(Arg1861*) | |  | |
| DE03M032722 | *MYO7A* | p.(Arg1240Gln) | p.(Arg1240Gln) | |  | |
| DE03M05340 | *MYO7A* | p.(Arg1240Gln) | p.(Arg1883Gln) | |  | |
| DE03M10501^#^ | *MYO7A* | p.(Glu1716*) | p.(Lys1255Argfs*8) | |  | |
| DE03M06650 | *MYO7A* | p.(Leu1858Pro) | p.(Arg1240Gln) | |  | |
| DE03M029991 | *MYO7A* | p.(Thr165Met) | p.(Glu968Asp) | |  | |
| IT02M1710060^#^ | *MYO7A* | **p.(Lys1308=)** | p.(Leu1836Pro) | |  | |
| IT02M071081 | *MYO7A* | p.(Arg241Gly) | p.(Ala26Glu) | |  | |
| IT02M181141 | *MYO7A* | **p.(Tyr2136*)** | p.(Ser1471Pro) | |  | |
| IT02M061161 | *MYO7A* | **p.(Gln1690*)** | p.(Gly25Arg) | |  | |
| IT02M041311 | *MYO7A* | **c.1691-4G>A** | **p.(Thr62Argfs*9)** | |  | |
| IT02M081610 | *MYO7A* | p.(Arg241Gly) | p.(Arg241Gly) | |  | |
| IT02M121661 | *MYO7A* | p.(Arg1861*) | p.(Arg1861*) | |  | |
| IT02M091720 | *MYO7A* | p.(Arg241Gly) | p.(Arg241Gly) | |  | |
| IT02M011730 | *MYO7A* | p.(Phe1946Serfs*23) | p.(Phe1946Serfs*23) | |  | |
| IT02M101740 | *MYO7A* | p.(Arg241Gly) | p.(Arg241Gly) | |  | |
| IT02M031781 | *MYO7A* | **p.(Gln1690*)** | **c.(6237+1_6238-1)_(6354+1_6355-1)del** | | p.(Gly2163Ser) | |
| IT02M0518421 | *MYO7A* | p.(Arg657Trp) | p.(Arg1873Trp) | |  | |
| IT02M161881 | *MYO7A* | c.1344-2A>G | c.1344-2A>G | |  | |
| IT02M141910 | *MYO7A* | p.(Arg669*) | p.(Glu166Argfs*5) | |  | |
| IT02M111920 | *MYO7A* | p.(Arg241Gly) | c.5856+1G>C | |  | |
| IT02M1321071 | *MYO7A* | **p.(Gln706*)** | p.(Arg1861*) | |  | |
| SLO01M1011152 | *MYO7A* | p.(Arg666*) | **p.(Ser1605Phe)** | |  | |
| SLO01M0811172 | *MYO7A* | p.(Leu366Pro) | p.(Leu366Pro) | |  | |
| SLO01M0111202 | *MYO7A* | p.(Arg1240Gln) | p.(Arg1240Gln) | |  | |
| SLO01M0311242 | *MYO7A* | p.(Gln18*) | p.(Arg2024*) | |  | |
| SLO01M0411302 | *MYO7A* | p.(Leu366Pro) | p.(Gln18*) | |  | |
| SLO01M0611342 | *MYO7A* | p.(Gln18*) | p.(Gln18*) | |  | |
| SLO01M0911412 | *MYO7A* | p.(Leu366Pro) | p.(Leu366Pro) | |  | |
| SLO01M0511552 | *MYO7A* | p.(Gln1088*) | p.(Gln18*) | |  | |
| SLO01M1111592 | *MYO7A* | **p.(Gln775*)** | **p.(Pro638His)** | |  | |
| SLO01M0212352 | *MYO7A* | p.(Gln18*) | p.(Arg1240Gln) | |  | |
| SLO01M0713052 | *MYO7A* | p.(Gln18*) | p.(Gln18*) | |  | |
| SP01M14010040531 | *MYO7A* | **p.(Arg1883Trp)** | **p.(Arg1883Trp)** | |  | |
| SP01M111023170 | *MYO7A* | p.(Gln1433Serfs*116) | p.(Ala2009Profs*32) | |  | |
| SP01M03107360 | *MYO7A* | c.2283-1G>T | p.(Cys1198*) | |  | |
| SP01M07126910 | *MYO7A* | p.(Tyr333*) | p.(Tyr333*) | |  | |
| SP01M05127011 | *MYO7A* | **p.(Glu739*)** | p.(Arg241His) | |  | |
| SP01M08127791 | *MYO7A* | p.(Tyr333*) | p.(Tyr333*) | |  | |
| SP01M061313811 | *MYO7A* | **p.(Gly1982Arg)** | **p.(Gly1982Arg)** | |  | |
| SP01M02135721 | *MYO7A* | p.(Leu4fsAsp*39) | p.(Leu1858Pro) | |  | |
| SP01M09141471 | *MYO7A* | p.(Tyr333*) | p.(Tyr333*) | |  | |
| SP01M121415951 | *MYO7A* | p.(Trp2107*) | p.(Ala2009Profs*32) | |  | |
| SP01M131419521 | *MYO7A* | p.(Arg972*) | p.(Gly1378Trpfs*6) | |  | |
| SP01M011428841 | *MYO7A* | p.(Gln1798*) | p.(Gln1798*) | |  | |
| SP01M151519262 | *MYO7A* | **p.(Tyr235Asp)** | **p.(Arg1883Trp)** | |  | |
| SP01M101520730 | *MYO7A* | p.(Tyr333*) | p.(Tyr333*) | |  | |
| SP01M17702202321 | *MYO7A* | p.(Glu1170Lys) | p.(Glu1170Lys) | |  | |
| SP01M16802261941 | *MYO7A* | p.(Gln959Glyfs*5) | p.(Gln959Glyfs*5) | |  | |
| SP01M04812011890 | *MYO7A* | **p.(Gly1497Arg)** | **c.(6237+1_6238-1)_(6354+1_6355-1)del** | |  | |
| FR02P0530112 | *PCDH15* | **p.(*1791Argext*5)** | **c.(?_-395)del** | |  | |
| FR02P0149982 | *PCDH15* | p.(Arg250*) | p.(Arg250*) | |  | |
| FR02P0463782 | *PCDH15* | **c.(?_-395)_(91+1_92-1)del** | **c.(?_-395)_(91+1_92-1)del** | |  | |
| FR02P03S48931 | *PCDH15* | **p.(Tyr662*)** | **c.(?_-395)_(91+1_92-1)del** | |  | |
| FR02P02S53991 | *PCDH15* | **c.107-2A>G** | p.(Arg341*) | |  | |
| DE03P01171 | *PCDH15* | **p.(Leu1390Valfs*26)** | **c.(1113+1_1114-1)_(1320+1_1321-1)del** | |  | |
| SLO01P0411182 | *PCDH15* | c.3753+1 G>A | p.(Leu368Trpfs*58) | |  | |
| SLO01P0111252 | *PCDH15* | p.(Leu368Trpfs*58) | p.(Leu368Trpfs*58) | |  | |
| SLO01P0211262 | *PCDH15* | p.(Leu368Trpfs*58) | p.(Leu368Trpfs*58) | |  | |
| SLO01P0311272 | *PCDH15* | p.(Leu368Trpfs*58) | p.(Leu368Trpfs*58) | |  | |
| SP01P0113161 | *PCDH15* | p.(Arg3*) | p.(Arg3*) | |  | |
| FR02H0136072 | *USH1C* | **p.(Arg196*)** | **p.(Arg196*)** | |  | |
| FR02H0314059740 | *USH1C* | p.(Arg80Profs*69) | **c.104+1G>A** | |  | |
| FR02H02S37330 | *USH1C* | p.(Arg155*) | p.(Arg155*) | |  | |
| FR02H04S60390 | *USH1C* | **p.(Lys12=)** | c.580-2A>T | |  | |
| FR02H05U1811 | *USH1C* | **p.(Gly829=)** | **p.(Arg620Leu)** | |  | |
| DE03H021130 | *USH1C* | **p.(Gln383Thrfs*6)** | **p.(Gln383Thrfs*6)** | |  | |
| DE03H03310 | *USH1C* | **c.388-1G>C** | **c.388-1G>C** | |  | |
| IT02H011890 | *USH1C* | p.(Arg80Profs*69) | p.(Arg80Profs*69) | |  | |
| IT02H021900 | *USH1C* | p.(Arg80Profs*69) | p.(Arg80Profs*69) | |  | |
| SLO01H0111162^#^ | *USH1C* | p.(Arg80Profs*69) | p.(Arg80Profs*69) | |  | |
| FR02S0157792 | *USH1G* | **p.(Gln248*)** | **p.(Gln248*)** | |  | |
| FR02S02P02571 | *USH1G* | p.(Trp38*) | **c.(?_-190)_(*1992_?)del** | |  | |
| DE03S01911 | *USH1G* | **p.(Lys130Glnfs*5)** | **p.(Lys130Glnfs*5)** | |  | |
| IT02S011851 | *USH1G* | **c.(1382+1_1383-1)_(*1992_?)del** | **c.(1382+1_1383-1)_(*1992_?)del** | |  | |

All missense mutations are predicted to be pathogenic. Novel mutations are indicated in bold.

FR = France; IT = Italy; SP = Spain; GER = Germany; SLO = Slovenia

^#^patients with phenotype/genotype discrepancy
