# Supplementary material for: An innovative strategy for the molecular diagnosis of Usher syndrome identifies causal biallelic mutations in 93% of European patients
Source: Eur J Hum Genet. 2016 Jul 27;24(12):1730–8. doi: 10.1038/ejhg.2016.99 (PMC5117943; doi:10.1038/ejhg.2016.99)
Supplement: Supplementary Table 2 [file ejhg201699x2.docx]

**Supplementary Table 2: Genotype of the USH2 and USH3 patients**

| **Patient Number** | **Gene** | **Mutation 1** | **Mutation 2** | **Additional mutation** |
| --- | --- | --- | --- | --- |
|  |  |  |  |  |
| FR02G011822 | *ADGRV1* | **p.(Gly2329Argfs*17)** | **c.9906+1G>A** |  |
| FR02G028272 | *ADGRV1* | **p.(Arg493*)** | **c.1509+3A>G** |  |
| FR02G0315122 | *ADGRV1* | **p.(Gln5626*)** | **p.(Gln5626*)** |  |
| FR02G0415382 | *ADGRV1* | p.(Glu4186Glyfs*17) | **c.6951+1_6952-1)_(7133+1_7134-1)del** |  |
| FR02G0534762 | *ADGRV1* | **p.(Gln3490*)** | **p.(Gln3490*)** |  |
| FR02G0636522 | *ADGRV1* | **p.(Ala2032Argfs*27)** | **c.9184+3A>G** |  |
| FR02G0746112 | *ADGRV1* | **p.(Ser4048*)** | p.(Asp3992Asn) |  |
| FR02G0850662 | *ADGRV1* | p.(Ser4441Leufs*9) | p.(Val5647Glyfs*7) |  |
| FR02G0963012 | *ADGRV1* | p.(Trp3486*) | **c.(3022+1_3023-1)_(16611+1_16612-1)dup** |  |
| FR02G1083212 | *ADGRV1* | p.(Ser955*) | p.(Ser955*) |  |
| FR02G1114024350 | *ADGRV1* | p.(Met5890Valfs*10) | p.(Met5890Valfs*10) |  |
| DE03G011211 | *ADGRV1* | **p.(Arg3293*)** | **p.(Ser1358Pro)** |  |
| DE03G021752 | *ADGRV1* | **p.(Gly2045*)** | **c.10054-1G>T** |  |
| DE03G03480 | *ADGRV1* | p.(Gln753Leufs*8) | p.(Lys1786Ilefs*8) |  |
| DE03G04551 | *ADGRV1* | **p.(Tyr536*)** | **p.(Tyr536*)** |  |
| DE03G05611 | *ADGRV1* | **p.(Phe5328Serfs*41)** | **p.(Glu2669Lysfs*4)** |  |
| DE03G06870 | *ADGRV1* | p.(Trp3486*) | **p.(Glu6295Alafs*31)** |  |
| IT02G012020 | *ADGRV1* | **p.(Leu568Cysfs*8)** | **p.(Cys5970Ser)** |  |
| DK01U02698401 | *USH2A* | **p.(Ala4423Asp)** | p.(Ser1136Asn) |  |
| DK01U01845151 | *USH2A* | p.(Glu767Serfs*21) | p.(Glu767Serfs*21) |  |
| DK01U03886370 | *USH2A* | **p.(Thr5006Met)** | p.(Arg2853*) |  |
| FR01U02A11551 | *USH2A* | **p.(Pro4232Leu )** | **c.(1550+1_1551-1)_(4627+1_4628-1)del** |  |
| FR01U03A1430 | *USH2A* | p.(Leu4282Pro) | p.(Leu4282Pro) |  |
| FR01U04ATG700 | *USH2A* | p.(Gln3587*) | **c.6657+1G>C** |  |
| FR02U05242 | *USH2A* | **p.(Ile1557Leufs*18)** | **c.3317-1G>A** |  |
| FR02U06392 | *USH2A* | p.(Tyr4128Hisfs*24) | p.(Glu767Serfs*21) |  |
| FR02U07472 | *USH2A* | **p.(Thr1443Pro)** | p.(Trp3955*) |  |
| FR02U08872 | *USH2A* | c.12067-2A>G | c.12067-2A>G |  |
| FR02U081112 | *USH2A* | c.785-6636_1840+208del | c.785-6636_1840+208del |  |
| FR02U091752 | *USH2A* | p.(Thr4439Ile) | p.(Arg4115Cys)/p.(Thr4425Met) |  |
| FR02U102062 | *USH2A* | p.(His308Serfs*16) | **c.(1644+1_1645-1)_(4627+1_4628-1)del** |  |
| FR02U113302 | *USH2A* | **p.(Phe4697Leufs*2)** | p.(Tyr4031*) |  |
| FR02U123922 | *USH2A* | p.(Arg4935*) | p.(His308Serfs*16) |  |
| FR02U135262 | *USH2A* | p.(Thr4809Ile) | p.(Glu767Serfs*21) |  |
| FR02U146642 | *USH2A* | p.(Gln919*) | p.(Glu767Serfs*21) |  |
| FR02U159252 | *USH2A* | p.(Arg998Lys) | p.(=,Tyr318Cysfs*17) |  |
| FR02U1610392 | *USH2A* | p.(Glu284Aspfs*38) | c.7595-2144A>G |  |
| FR02U1713932 | *USH2A* | **p.(Tyr2900Asn )** | **p.(Gly1668Argfs*30)** |  |
| FR02U1815092 | *USH2A* | **p.(Pro4735Arg )** | c.12067-2A>G |  |
| FR02U1915842 | *USH2A* | p.(Val218Glu) | p.(Glu767Serfs*21) |  |
| FR02U2016832 | *USH2A* | p.(Arg626*) | p.(Cys759Phe) |  |
| FR02U2119962 | *USH2A* | **p.(Leu1317*)** | p.(Glu767Serfs*21) |  |
| FR02U2225512 | *USH2A* | **p.(Glu287*)** | p.(Asn346His) |  |
| FR02U2327632 | *USH2A* | c.3317-2A>G | p.(Glu767Serfs*21) |  |
| FR02U2429002 | *USH2A* | **p.(Gly4095Ser)** | p.(Glu767Serfs*21) |  |
| FR02U2529982 | *USH2A* | **p.(Pro5127Argfs*8)** | **p.(Pro5127Argfs*8)** |  |
| FR02U2730432 | *USH2A* | p.(Ser1247Lysfs*4) | c.9371+1G>T |  |
| FR02U2830812 | *USH2A* | **p.(Gly4095Ser)** | p.(Cys759Phe) |  |
| FR02U2934662 | *USH2A* | **p.(Gly3264Valfs*37)** | p.(Arg5143His) |  |
| FR02U3036172 | *USH2A* | **p.(Tyr4414Cysfs*12)** | p.(Glu767Serfs*21) | c.11548+2T>G |
| FR02U3137132 | *USH2A* | p.(Glu2288*) | p.(Thr4999Ile) |  |
| FR02U3241292 | *USH2A* | p.(Arg2509Glyfs*19) | p.(Trp3955*) |  |
| FR02U3342772 | *USH2A* | **p.(Gly1841Glu)** | p.(Gly268Arg) |  |
| FR02U3443182 | *USH2A* | **p.(Ala1711Serfs*6)** | p.(Glu767Serfs*21) |  |
| FR02U3545182 | *USH2A* | p.(Cys5122Arg) | p.(Arg1653*) |  |
| FR02U3646012 | *USH2A* | p.(Leu4282Pro) | p.(Cys759Phe) |  |
| FR02U3750672 | *USH2A* | c.13811+2T>G | p.(Pro3272Leu) |  |
| FR02U2651202 | *USH2A* | p.(His308Serfs*16) | p.(Asn346His) |  |
| FR02U3851612 | *USH2A* | p.(Arg737*) | **c.11389+3A>G** |  |
| FR02U3953202 | *USH2A* | p.(Gly660Arg) | p.(Val218Glu) |  |
| FR02U4054352 | *USH2A* | p.(Cys419Phe) | **p.(Ile5166Val)** |  |
| FR02U4155612 | *USH2A* | p.(Cys419Phe) | **p.(Tyr4298Cys)** |  |
| FR02U4256182 | *USH2A* | p.(Arg34*) | p.(Arg3719His) |  |
| FR02U4357322 | *USH2A* | p.(Arg34*) | p.(Glu767Serfs*21) |  |
| FR02U4457522 | *USH2A* | p.(Val2244Met) | p.(Leu4567Profs*16) |  |
| FR02U4559922 | *USH2A* | p.(Glu767Serfs*21) | p.(Val218Glu) |  |
| FR02U4660482 | *USH2A* | c.7595-3C>G | c.7595-2144A>G |  |
| FR02U4761072 | *USH2A* | p.(Thr4439Ile) | p.(Glu767Serfs*21) |  |
| FR02U4861552 | *USH2A* | **p.(Asn4101del)** | c.14791+5G>T |  |
| FR02U4961592 | *USH2A* | p.(Arg626*) | p.(Glu767Serfs*21) |  |
| FR02U5061812 | *USH2A* | p.(Thr4337Met) | p.(Cys717Gly) |  |
| FR02U5163062 | *USH2A* | p.(Glu767Serfs*21) | p.(Thr4337Met) |  |
| FR02U0163292 | *USH2A* | p.(Glu767Serfs*21) | p.(Glu767Serfs*21) |  |
| FR02U5267932 | *USH2A* | p.(Glu767Serfs*21) | p.(Gln1408*) |  |
| FR02U5368522 | *USH2A* | p.(Glu767Serfs*21) | **p.(Val1816Gly)** |  |
| FR02U5470382 | *USH2A* | **p.(Thr3494Arg)** | p.(Arg4935*) |  |
| FR02U5570582 | *USH2A* | **p.(Glu3395*)** | p.(Arg4608*) |  |
| FR02U5676022 | *USH2A* | p.(Cys759Phe) | p.(Arg1549*) |  |
| FR02U5776512 | *USH2A* | p.(Trp3955*) | p.(Glu4458Aspfs*3) |  |
| FR02U5876892 | *USH2A* | p.(Glu767Serfs*21) | **p.(Thr5006Met)** |  |
| FR02U5981182 | *USH2A* | p.(Cys870*) | p.(Trp3955*) |  |
| FR02U6081452 | *USH2A* | p.(Glu767Serfs*21) | p.(Cys766Arg) |  |
| FR02U6181962 | *USH2A* | p.(Ser5030*) | p.(Cys934Trp) |  |
| FR02U6213037171 | *USH2A* | p.(Arg4935*) | p.(Thr4809Ile) |  |
| FR02U6313037701 | *USH2A* | p.(Glu767Serfs*21) | c.(4251+1_4252-1)_(4396+1_4397-1)del |  |
| FR02U6413045260 | *USH2A* | p.(Trp4725*) | p.(His308Serfs*16) |  |
| FR02U6513049330 | *USH2A* | p.(Leu1572Phefs*3) | c.7595-2144A>G |  |
| FR02U6614002801 | *USH2A* | **c.9056-1_9056delinsTC** | p.(Cys691Arg) |  |
| FR02U6714057401 | *USH2A* | p.(Glu767Serfs*21) | p.(Val4745Argfs*4) |  |
| FR02U68P04181 | *USH2A* | p.(Ser5030*) | **c.(6658+1_6659-1)_(6805+1_6806-1)del** |  |
| FR02U69S14220 | *USH2A* | p.(Glu284Aspfs*38) | **p.(Cys696Ser)** |  |
| FR02U70S21420 | *USH2A* | p.(Arg626*) | p.(Arg626*) | *USH1G* **p.(Trp267*)** |
| FR02U71S27881 | *USH2A* | p.(Glu767Serfs*21) | **c.(6163+1_6164-1)_(6325+1_6326-1)del** |  |
| FR02U72S57220 | *USH2A* | p.(Pro1843Leu) | p.(Trp3955*) |  |
| FR02U73U1020 | *USH2A* | **p.(Glu4963Glyfs*38)** | c.4758+1G>A |  |
| FR02U74U1060 | *USH2A* | **p.(Gly4817Glu)** | p.(Cys3267Arg) |  |
| FR02U75U1540 | *USH2A* | **c.(651+1_652-1)_(784+1_785-1)del** | **p.(Ser3005Thr)** | *CDH23* **c.(336+1_337-1)_(4617+1_4618-1)dup** |
| FR02U76U1651 | *USH2A* | p.(Ala3660fsVal*14) | p.(Gln3959Asnfs*53) |  |
| FR02U77U1680 | *USH2A* | **p.(Lys811*)** | c.7595-2144A>G |  |
| FR02U78U1870 | *USH2A* | p.(=,Tyr318Cysfs*17) | p.(Arg4115Cys)/p.(Thr4425Met) |  |
| FR02U79U1931 | *USH2A* | p.(Ala3660fsVal*14) | c.7595-2144A>G |  |
| FR02U80U1990 | *USH2A* | **p.(Tyr188Asn)** | **p.(Arg274Gln)** |  |
| FR02U81U2181 | *USH2A* | p.(Pro3116Hisfs*13) | p.(Pro3116Hisfs*13) |  |
| FR02U82U2210 | *USH2A* | p.(Glu511Lys ) | p.(Asn4079Trpfs*19) |  |
| FR02U83U2220 | *USH2A* | p.(Leu2319Argfs*7) | p.(Glu767Serfs*21) | p.(Asp3288His) |
| FR02U84U2340 | *USH2A* | p.(His308Serfs*16) | p.(Glu767Serfs*21) |  |
| FR02U85U2531 | *USH2A* | p.(Leu4840Pro) **p.(Cys1900Gly)** | p.(Leu4840Pro) **p.(Cys1900Gly)** |  |
| FR02U86U2561 | *USH2A* | p.(Thr4439Ile) | c.7595-2144A>G |  |
| FR02U87U2591 | *USH2A* | p.(Thr4425Met)/p.(Arg4115Cys) | p.(Thr4425Met)/p.(Arg4115Cys) |  |
| FR02U88U2610 | *USH2A* | p.(Glu767Serfs*21) | **p.(Tyr3940Cys)** |  |
| FR02U89U2631 | *USH2A* | p.(Cys3267Arg) | **c.486-2A>C** |  |
| FR02U90U700 | *USH2A* | p.(Glu767Serfs*21) | p.(Trp3702*) |  |
| FR02U91U720 | *USH2A* | **p.(Gln4635*)** | **p.(Ile4059Asnfs*40)** |  |
| FR02U92U741 | *USH2A* | p.(Pro3116Hisfs*13) | p.(Thr4337Met) |  |
| FR02U93U920 | *USH2A* | **p.(Val2715*)** | c.7595-2144A>G |  |
| FR02U94U991 | *USH2A* | p.(Ser3856Valfs*28) | p.(Glu767Serfs*21) |  |
| DE03U06091 | *USH2A* | **p.(Cys536*)** | p.(Glu767Serfs*21) |  |
| DE03U07100 | *USH2A* | p.(Arg626*) | **p.(Gly516Arg)** |  |
| DE03U081001 | *USH2A* | p.(Cys520Arg) | p.(Cys520Arg) |  |
| DE03U091011 | *USH2A* | p.(Trp3521Arg) | p.(Glu767Serfs*21) |  |
| DE03U101020 | *USH2A* | p.(Trp2945*) | p.(Cys2128Tyr) |  |
| DE03U111041 | *USH2A* | p.(Gln4711*) | p.(Trp3521Arg) |  |
| DE03U121140 | *USH2A* | p.(Asn4079Trpfs*19) | p.(Asn4079Trpfs*19) |  |
| DE03U13121 | *USH2A* | p.(Arg3538*) | p.(Glu767Serfs*21) |  |
| DE03U141201 | *USH2A* | p.(Arg4935*) | p.(Gly3142*) |  |
| DE03U011240 | *USH2A* | p.(Glu767Serfs*21) | p.(Glu767Serfs*21) |  |
| DE03U151250 | *USH2A* | p.(Tyr1103*) | **p.(His340Asp)** |  |
| DE03U161282 | *USH2A* | p.(Glu767Serfs*21) | p.(Gln4235*) |  |
| DE03U171292 | *USH2A* | p.(Trp3955*) | **c.(11548+1_11549-1)_(11711+1_11712-1)del** |  |
| DE03U181302 | *USH2A* | p.(Cys3090*) | p.(Trp3955*) |  |
| DE03U191312 | *USH2A* | c.6805+2T>C | p.(Arg334Trp) |  |
| DE03U201342 | *USH2A* | **p.(Trp2841*)** | **c.9258+1G>A** |  |
| DE03U21141 | *USH2A* | p.(Glu767Serfs*21) | **p.(Ile3103Val)** |  |
| DE03U221482 | *USH2A* | **c.1144-2A>T** | p.(Trp3955*) |  |
| DE03U231492 | *USH2A* | p.(Val218Glu) | p.(Glu767Serfs*21) |  |
| DE03U241502 | *USH2A* | p.(Val218Glu) | p.(Trp3955*) |  |
| DE03U25161 | *USH2A* | p.(Val218Glu) | p.(Cys419Phe) |  |
| DE03U26290 | *USH2A* | **p.(Cys4140Phe)** | p.(Glu4458Aspfs*3) | **p.(Ala3865Glu)** |
| DE03U27321 | *USH2A* | p.(=,Tyr318Cysfs*17) | **p.(Ile5166Val)** |  |
| DE03U28360 | *USH2A* | p.(Gly3647Ser) | p.(Leu2380Profs*37) |  |
| DE03U29381 | *USH2A* | p.(Ala3944Asp) | c.5776+1G>A |  |
| DE03U30390 | *USH2A* | p.(Trp2945*) | c.4627+25435_4987+660del |  |
| DE03U31401 | *USH2A* | p.(Glu767Serfs*21) | **c.(3157+1_3158-1)_(4627+1_4628-1)del** |  |
| DE03U32410 | *USH2A* | p.(Cys870*) | **c.(2993+1_2994-1)_(3157+1_3158-1)del** |  |
| DE03U33461 | *USH2A* | p.(Ser259Phefs*63) | p.(Trp3955*) |  |
| DE03U34490 | *USH2A* | c.11549-1G>A | p.(Glu767Serfs*21) |  |
| DE03U35510 | *USH2A* | p.(Gln4711*) | **c.5776+2T>C** |  |
| DE03U36530 | *USH2A* | **p.(Trp2075Cys)** | p.(Glu767Serfs*21) |  |
| DE03U37541 | *USH2A* | p.(Trp3521Arg) | p.(Glu767Serfs*21) |  |
| DE03U38561 | *USH2A* | p.(Cys2309Phe) | p.(Glu767Serfs*21) |  |
| DE03U02581 | *USH2A* | p.(Glu767Serfs*21) | p.(Glu767Serfs*21) |  |
| DE03U05630 | *USH2A* | p.(Trp3955*) | p.(Trp3955*) |  |
| DE03U39661 | *USH2A* | p.(Arg4192Cys) | p.(Cys870*) |  |
| DE03U40671 | *USH2A* | p.(Glu767Serfs*21) | c.7595-2144A>G |  |
| DE03U41701 | *USH2A* | p.(Glu767Serfs*21) | **c.(11548+1_11549-1)_(11711+1_11712-1)del** |  |
| DE03U42710 | *USH2A* | p.(=,Tyr318Cysfs*17) | p.(Trp3955*) |  |
| DE03U43730 | *USH2A* | c.11549-1G>A | **c.(2993+1_2994-1)_(13811+1_13812-1)dup** |  |
| DE03U44750 | *USH2A* | p.(Trp3702*) | p.(Trp3702*) |  |
| DE03U45770 | *USH2A* | **c.5777-2A>G** | p.(Trp3521Arg) |  |
| DE03U03800 | *USH2A* | p.(Glu767Serfs*21) | p.(Glu767Serfs*21) |  |
| DE03U46811 | *USH2A* | p.(Asn346His) | p.(Trp3955*) |  |
| DE03U47821 | *USH2A* | p.(Thr2919Pro) | p.(Cys870*) |  |
| DE03U48840 | *USH2A* | **c.14791+2T>C** | c.9259-2402_9371+1537del |  |
| DE03U49851 | *USH2A* | p.(Trp3702*) | p.(Trp3702*) |  |
| DE03U04931 | *USH2A* | p.(Glu767Serfs*21) | p.(Glu767Serfs*21) |  |
| DE03U50950 | *USH2A* | **p.(Asn2651Glnfs*10)** | p.(=,Tyr318Cysfs*17) |  |
| DE03U51960 | *USH2A* | p.(Glu4458Aspfs*3) | p.(Glu767Serfs*21) |  |
| DE03U52980 | *USH2A* | **p.(Gln4676Profs*7)** | p.(Trp3955*) |  |
| DE03U53990 | *USH2A* | p.(Trp3521Arg) | c.785-6636_1840+208del |  |
| DE03U549981 | *USH2A* | p.(Trp3955*) | p.(Met1863Thr) |  |
| DE03U5519602 | *USH2A* | p.(Trp3955*) | p.(Arg303His) |  |
| IT02U0110011 | *USH2A* | **p.(Cys953*)** | p.(Trp3955*) |  |
| IT02U0210041 | *USH2A* | **p.(Asn330Lysfs*8)** | p.(Thr3571Met) |  |
| IT02U0310050 | *USH2A* | **c.14791+4A>G** | p.(Thr3571Met) |  |
| IT02U0410110 | *USH2A* | p.(Ile285Thr) | p.(Arg737*) |  |
| IT02U0510130 | *USH2A* | **p.(Ser1350Leufs*16)** | p.(Gly268Arg) |  |
| IT02U0610141 | *USH2A* | p.(Leu3606Pro) | c.1841-2A>G |  |
| IT02U0710160 | *USH2A* | **p.(Gln1573*)** | p.(Thr3571Met) |  |
| IT02U0810602 | *USH2A* | p.(Phe4993Profs*7) | p.(Phe4993Profs*7) |  |
| IT02U091070 | *USH2A* | **p.(Pro2241Thr)** | p.(Glu767Serfs*21) |  |
| IT02U1012180 | *USH2A* | p.(Thr352Ile) | p.(Thr3571Met) |  |
| IT02U111320 | *USH2A* | **p.(Trp1084*)** | p.(Glu767Serfs*21) |  |
| IT02U1214361 | *USH2A* | **p.(Tyr3715*)** | p.(Thr3571Met) |  |
| IT02U1314641 | *USH2A* | **p.(Pro309Leu)** | p.(Glu767Serfs*21) |  |
| IT02U1414820 | *USH2A* | **p.(Trp211*)** | **p.(Gly4340Arg)** |  |
| IT02U1515091 | *USH2A* | p.(Phe4993Profs*7) | **p.(Tyr90Cys)** |  |
| IT02U1615200 | *USH2A* | p.(Trp3955*) | p.(Glu767Serfs*21) |  |
| IT02U1715361 | *USH2A* | **p.(Gln4750*)** | p.(Met3271Cysfs*30) |  |
| IT02U1816640 | *USH2A* | **p.(Ser2969*)** | **p.(Ser2969*)** |  |
| IT02U1916760 | *USH2A* | **p.(Tyr4329*)** | p.(Gly516Val) |  |
| IT02U2017672 | *USH2A* | **c.7452-1G>A** | **c.7452-1G>A** |  |
| IT02U2118741 | *USH2A* | **p.(Glu2242*)** | p.(Arg303His) |  |
| IT02U2219552 | *USH2A* | **p.(Tyr2566Leufs*11)** | **p.(Arg3119Cys)** |  |
| IT02U2319930 | *USH2A* | **p.(Val2700Glu)** | p.(Glu1492*) |  |
| IT02U2420650 | *USH2A* | p.(Phe4993Profs*7) | p.(Phe4993Profs*7) |  |
| IT02U2521551 | *USH2A* | **p.(Cys1228*)** | p.(Thr3571Met) |  |
| IT02U262321 | *USH2A* | p.(Ser2639Pro) | p.(Ser2639Pro) |  |
| IT02U272481 | *USH2A* | p.(Trp3955*) | **c.(3316+1_3317-1)_(4627+1_4628-1)del** |  |
| IT02U282841 | *USH2A* | p.(Thr3571Met) | p.(Thr3571Met) |  |
| IT02U293311 | *USH2A* | p.(Arg2509Glyfs*19) | p.(Arg1777Trp) |  |
| IT02U304071 | *USH2A* | **p.(Glu4963Glyfs*38)** | p.(Trp3955*) |  |
| IT02U31410 | *USH2A* | p.(Thr3571Met) | p.(Thr3571Met) |  |
| IT02U32601 | *USH2A* | **p.(Phe1297Serfs*17)** | c.(4251+1_4252-1)_(4396+1_4397-1)del |  |
| IT02U337021 | *USH2A* | **p.(Phe1297Serfs*17)** | p.(Thr3571Met) |  |
| IT02U348231 | *USH2A* | **p.(Phe1297Serfs*17)** | p.(Thr3571Met) |  |
| SLO01U1110012 | *USH2A* | p.(Cys870*) | p.(Trp3955*) |  |
| SLO01U1211052 | *USH2A* | **p.(Gly1645*)** | p.(Trp3955*) |  |
| SLO01U1311062 | *USH2A* | **p.(Phe78Val)** | p.(Trp3955*) |  |
| SLO01U1411072 | *USH2A* | c.4627+25435_4987+660del | c.4627+25435_4987+660del |  |
| SLO01U1511102 | *USH2A* | p.(Cys870*) | p.(Trp3955*) |  |
| SLO01U1611132 | *USH2A* | p.(Thr352Ile) | p.(Trp3955*) |  |
| SLO01U0111142 | *USH2A* | p.(Trp3955*) | p.(Trp3955*) |  |
| SLO01U1711192 | *USH2A* | p.(Trp3955*) | **p.(Thr3676Ile)** |  |
| SLO01U1811212 | *USH2A* | **p.(Gly1645*)** | p.(Trp3955*) |  |
| SLO01U0211322 | *USH2A* | p.(Trp3955*) | p.(Trp3955*) |  |
| SLO01U0311332 | *USH2A* | p.(Trp3955*) | p.(Trp3955*) |  |
| SLO01U1911352 | *USH2A* | p.(Trp3955*) | p.(Gly4763Arg) |  |
| SLO01U2011362 | *USH2A* | p.(Trp3955*) | p.(Arg2914*) |  |
| SLO01U2111392 | *USH2A* | p.(Trp3955*) | p.(Gly4095Asp) |  |
| SLO01U2211422 | *USH2A* | p.(Trp3955*) | p.(Arg303His) | p.(Gly3618Ser) |
| SLO01U2311432 | *USH2A* | p.(Trp3955*) | p.(Gln3292*) |  |
| SLO01U0411442 | *USH2A* | p.(Trp3955*) | p.(Trp3955*) |  |
| SLO01U2411452 | *USH2A* | p.(Trp3955*) | p.(Arg303His) |  |
| SLO01U2511542 | *USH2A* | **p.(Thr4315Pro)** | p.(Cys870*) |  |
| SLO01U2611562 | *USH2A* | p.(Cys870*) | p.(Trp3955*) |  |
| SLO01U2711572 | *USH2A* | p.(Arg1549*) | p.(Trp3955*) |  |
| SLO01U2811582 | *USH2A* | **p.(Gly4857Ala)** | **c.(784+1_785-1)_(848+1_849-1)del** |  |
| SLO01U2912142 | *USH2A* | p.(Arg626*) | p.(Trp3955*) |  |
| SLO01U3012252 | *USH2A* | p.(Arg626*) | p.(Trp3955*) |  |
| SLO01U3112272 | *USH2A* | p.(Cys870*) | p.(Cys870*) |  |
| SLO01U0512362 | *USH2A* | p.(Trp3955*) | p.(Trp3955*) |  |
| SLO01U0612372 | *USH2A* | p.(Trp3955*) | p.(Trp3955*) |  |
| SLO01U3212512 | *USH2A* | **p.(Phe78Val)** | p.(Arg626*) |  |
| SLO01U0712562 | *USH2A* | p.(Trp3955*) | p.(Trp3955*) |  |
| SLO01U3312582 | *USH2A* | p.(Trp3955*) | **p.(Ala1241Valfs*8)** |  |
| SLO01U3412612 | *USH2A* | **p.(Pro2241His)** | p.(Trp3955*) |  |
| SLO01U3512672 | *USH2A* | p.(Cys870*) | p.(Cys870*) |  |
| SLO01U3612722 | *USH2A* | p.(Arg303His) | p.(Cys870*) |  |
| SLO01U0812792 | *USH2A* | p.(Trp3955*) | p.(Trp3955*) |  |
| SLO01U3712832 | *USH2A* | p.(Trp3955*) | **p.(Gly4032Arg)** |  |
| SLO01U3812852 | *USH2A* | p.(Trp3955*) | p.(Arg303His) |  |
| SLO01U0912882 | *USH2A* | p.(Trp3955*) | p.(Trp3955*) |  |
| SLO01U3913162 | *USH2A* | p.(Trp3955*) | p.(Asn4079Trpfs*19) |  |
| SLO01U4014012 | *USH2A* | p.(Trp3955*) | p.(Glu4458Aspfs*3) |  |
| SLO01U10STG02062 | *USH2A* | p.(Trp3955*) | p.(Trp3955*) |  |
| SP01U01107682 | *USH2A* | p.(Glu767Serfs*21) | c.7595-2144A>G |  |
| FR02CL1882 | *CLRN1* | **p.(Ala78Serfs*52)** | **p.(Ala78Serfs*52)** |  |
| FR02CL226442 | *CLRN1* | **p.(Gly59Valfs*13)** | p.(Asn48Lys) |  |
| FR02CL330052 | *CLRN1* | p.(Ala123Asp) | p.(Ala123Asp) |  |
| FR02CL454772 | *CLRN1* | p.(Ala123Asp) | p.(Ala123Asp) |  |
| FR02CL514036371 | *CLRN1* | **p.(Leu76Phefs*54)** | **p.(Leu76Phefs*54)** |  |
| FR02CL6S51931 | *CLRN1* | **p.(Gly77Glu)** | **p.(Gly77Glu)** |  |
| DE03CL21332 | *CLRN1* | **p.(Tyr100*)** | **p.(Tyr100*)** |  |
| DE03CL1741 | *CLRN1* | p.(Ile168Asnfs*5) | p.(Ser50Leufs*23) |  |

All missense mutations are predicted to be pathogenic. Novel mutations are indicated in bold.

FR = France; IT = Italy; SP = Spain; DEN = Denmark; GER = Germany; SLO = Slovenia
