# Supplementary material for: An innovative strategy for the molecular diagnosis of Usher syndrome identifies causal biallelic mutations in 93% of European patients
Source: Eur J Hum Genet. 2016 Jul 27;24(12):1730–8. doi: 10.1038/ejhg.2016.99 (PMC5117943; doi:10.1038/ejhg.2016.99)
Supplement: Supplementary Table 3 [file ejhg201699x3.docx]

| **Patient Number** | **Gene** | **Mutation 1** |
| --- | --- | --- |
| FR02C086142 | *CDH23* | **p.(Ile2132Metfs*11)** |
| FR02C0978842^#^ | *CDH23* | **dup exons 6-12** |
| FR02C10S30321 | *CDH23* | **c.7482+1G>A** |
| DE03C01941^#^ | *CDH23* | c.2289+1G>A |
| FR02M1479462 | *MYO7A* | p.(Phe1963del) |
| DE03M112772 | *MYO7A* | p.(Arg666*) |
| DE03M07891^#^ | *MYO7A* | p.(Ala2009Profs*32) |
| IT02M151800 | *MYO7A* | p.(Ala26Glu) |
| IT02M021831 | *MYO7A* | p.(Thr165Met) |
| SLO01M1211522 | *MYO7A* | **p.(Lys1737Glnfs*28)** |
| IT02P014170 | *PCDH15* | **del exons 15-18** |
| DE03H01641 | *USH1C* | p.(Arg80Profs*69) |
| FR02U9514552 | *USH2A* | p.(=,Tyr318Cysfs*17) |
| FR02U9644572 | *USH2A* | **p.(Gln4301Serfs*4)** |
| FR02U9765722 | *USH2A* | p.(Trp3955*) |
| FR02U9871892 | *USH2A* | p.(Glu767Serfs*21) |
| FR02U9978302 | *USH2A* | del exons 22-24 |
| FR03U0182112 | *USH2A* | **del exons 24-27** |
| IT02U3512230 | *USH2A* | c.1841-2A>G |
| IT02U362620 | *USH2A* | p.(Tyr4318*) |
| FR02G1230032 | *ADGRV1* | **p.(Arg6009*)** |
| FR02G13U1600 | *ADGRV1* | **p.(Phe3510Leufs*28)** |
| DE03G072602 | *ADGRV1* | **del exons 21-75** |
| FR02CL776062 | *CLRN1* | **c.434-2A>T** |
| IT02PZ15621 | *PDZD7* | **p.(Arg936*)** |

**Supplementary Table 3: Distribution of patients with only a partial diagnosis at the end of the study**

Novel mutations are indicated in bold.

^#^patients with phenotype/genotype discrepancy
