# Supplementary material for: An innovative strategy for the molecular diagnosis of Usher syndrome identifies causal biallelic mutations in 93% of European patients
Source: Eur J Hum Genet. 2016 Jul 27;24(12):1730–8. doi: 10.1038/ejhg.2016.99 (PMC5117943; doi:10.1038/ejhg.2016.99)
Supplement: Supplementary Table 4 [file ejhg201699x4.docx]

**Supplementary Table 4: Complete list of variants of predicted functional significance identified in this study**

| Gene | Nucleotide change | Predicted protein change |  |
| --- | --- | --- | --- |
| *MYO7A* | c.6_9dupGATT | p.(Leu4fsAsp*39) |  |
| (NG_009086.1) | c.52C>T | p.(Gln18*) |  |
| (NM_000260.3) | c.73G>A | p.(Gly25Arg) |  |
|  | c.77C>A | p.(Ala26Glu) |  |
|  | c.133-2A>G |  |  |
|  | **c.183delC** | **p.(Thr62Argfs*9)** |  |
|  | **c.262C>T** | **p.(Arg88Cys)** |  |
|  | c.397dupC | p.(His133Profs*7) |  |
|  | c.448C>T | p.(Arg150*) |  |
|  | c.487G>A | p.(Gly163Arg) |  |
|  | c.494C>T | p.(Thr165Met) |  |
|  | c.496delG | p.(Glu166Argfs*5) |  |
|  | c.592G>A | p.(Ala198Thr) |  |
|  | c.635G>A | p.(Arg212His) |  |
|  | c.640G>A | p.(Gly214Arg) |  |
|  | c.700C>T | p.(Gln234*) |  |
|  | **c.703T>G** | **p.(Tyr235Asp)** |  |
|  | c.721C>G | p.(Arg241Gly) |  |
|  | c.722G>A | p.(Arg241His) |  |
|  | c.805_807delAAG | p.(Lys269del) |  |
|  | c.999T>G | p.(Tyr333*) |  |
|  | c.1097T>C | p.(Leu366Pro) |  |
|  | c.1258A>T | p.(Lys420*) |  |
|  | **c.1307T>C** | **p.(Leu436Pro)** |  |
|  | c.1344-2A>G |  |  |
|  | **c.1580A>G** | **p.(Lys527Arg)** |  |
|  | c.1690+1G>A |  |  |
|  | **c.1691-4G>A** |  |  |
|  | **c.1717delC** | **p.(Leu573Cysfs*49)** |  |
|  | **c.1798-1G>A** |  |  |
|  | **c.1913C>A** | **p.(Pro638His)** |  |
|  | c.1969C>T | p.(Arg657Trp) |  |
|  | **c.1997G>A** | **p.(Arg666Gln)** |  |
|  | c.1996C>T | p.(Arg666*) |  |
|  | c.2005C>T | p.(Arg669*) |  |
|  | **c.2055C>A** | **p.(Tyr685*)** |  |
|  | **c.2116C>T** | **p.(Gln706*)** |  |
|  | **c.2210A>G** | **p.(Glu737Gly)** |  |
|  | **c.2215G>T** | **p.(Glu739*)** |  |
|  | c.2283-1G>T |  |  |
|  | **c.2323C>T** | **p.(Gln775*)** |  |
|  | c.2476G>A | p.(Ala826Thr) |  |
|  | c.2695-9A>G |  |  |
|  | c.2863G>A | p.(Gly955Ser) |  |
|  | c.2874_2878delCCAGG | p.(Gln959Glyfs*5) |  |
|  | c.2904G>C | p.(Glu968Asp) |  |
|  | c.2914C>T | p.(Arg972*) |  |
|  | c.3262C>T | p.(Gln1088*) |  |
|  | **c.3437G>A** | **p.(Arg1146Gln)** |  |
|  | **c.3470T>A** | **p.(Ile1157Asn)** |  |
|  | c.3503G>A | p.(Arg1168Gln) |  |
|  | c.3508G>A | p.(Glu1170Lys) |  |
|  | **c.3527G>A** | **p.(Ser1176Asn)** |  |
|  | **c.3546C>A** | **p.(Asn1182Lys)** |  |
|  | c.3594C>A | p.(Cys1198*) |  |
|  | c.3719G>A | p.(Arg1240Gln) |  |
|  | **c.3728C>T** | **p.(Pro1243Leu)** |  |
|  | c.3764delA | p.(Lys1255Argfs*8) |  |
|  | **c.3878_3879delTC** | **p.(Leu1293Glnfs*14)** |  |
|  | **c.3924G>A** | **p.(Lys1308=)** |  |
|  | c.4036_4038delTTC | p.(Phe1346del) |  |
|  | c.4131dupT | p.(Gly1378Trpfs*6) |  |
|  | **c.4159dupG** | **p.(Asp1387Glyfs*4)** |  |
|  | c.4297delC | p.(Gln1433Serfs*116) |  |
|  | c.4323+2T>G |  |  |
|  | c.4411T>C | p.(Ser1471Pro) |  |
|  | **c.4489G>C** | **p.(Gly1497Arg)** |  |
|  | **c.4814C>T** | **p.(Ser1605Phe)** |  |
|  | **c.4910_4911delAT** | **p.(His1637Argfs*17)** |  |
|  | **c.5000T>A** | **p.(Val1667Glu)** |  |
|  | **c.5068C>T** | **p.(Gln1690*)** |  |
|  | c.5146G>T | p.(Glu1716*) |  |
|  | **c.5168G>C** | **p.(Arg1723Thr)** |  |
|  | **c.5169-3G>C** |  |  |
|  | **c.5169-2A>G** |  |  |
|  | **c.5208dupC** | **p.(Lys1737Glnfs*28)** |  |
|  | **c.5215C>T** | **p.(Arg1739*)** |  |
|  | **c.5327-14T>G** |  |  |
|  | c.5392C>T | p.(Gln1798*) |  |
|  | c.5507T>C | p.(Leu1836Pro) |  |
|  | **c.5510T>A** | **p.(Leu1837His)** |  |
|  | c.5573T>C | p.(Leu1858Pro) |  |
|  | c.5581C>T | p.(Arg1861*) |  |
|  | c.5617C>T | p.(Arg1873Trp) |  |
|  | **c.5647C>T** | **p.(Arg1883Trp)** |  |
|  | c.5648G>A | p.(Arg1883Gln) |  |
|  | **c.5747delT** | **p.(Phe1916Serfs*54)** |  |
|  | c.5824G>A | p.(Gly1942Arg) |  |
|  | c.5835_5838delCTTT | p.(Phe1946Serfs*23) |  |
|  | c.5856+1G>C |  |  |
|  | c.5886_5888delCTT | p.(Phe1963del) |  |
|  | c.5899C>T | p.(Arg1967*) |  |
|  | **c.5916G>A** | **p.(Trp1972*)** |  |
|  | **c.5944G>A** | **p.(Gly1982Arg)** |  |
|  | c.6025delG | p.(Ala2009Profs*32) |  |
|  | **c.6025G>A** | **p.(Ala2009Thr)** |  |
|  | c.6028G>A | p.(Asp2010Asn) |  |
|  | c.6070C>T | p.(Arg2024*) |  |
|  | **c.6316A>T** | **p.(Lys2106*)** |  |
|  | c.6321G>A | p.(Trp2107*) |  |
|  | **c.6408T>G** | **p.(Tyr2136*)** |  |
|  | c.6487G>A | p.(Gly2163Ser) |  |
|  | **c.6439-1G>A** |  |  |
|  | c.6557T>C | p.(Leu2186Pro) |  |
|  | **c.(6237+1_6238-1)_(6354+1_6355-1)del** | **del exon 46** |  |
| *USH1C* | **c.36G>A** | **p.(Lys12=)** |  |
| (NG_011883.1) | **c.104+1G>A** |  |  |
| (NM_153676.3) | c.238dupC | p.(Arg80Profs*69) |  |
|  | **c.388-1G>C** |  |  |
|  | c.463C>T | p.(Arg155*) |  |
|  | c.580-2A>T |  |  |
|  | **c.586C>T** | **p.(Arg196*)** |  |
|  | **c.1146dupA** | **p.(Gln383Thrfs*6)** |  |
|  | **c.1859G>T** | **p.(Arg620Leu)** |  |
|  | **c.2487C>T** | **p.(Gly829=)** |  |
| *CDH23* | **c.122dupT** | **p.(Ile42Aspfs*22)** |  |
| (NG_008835.1) | **c.478G>A** | **p.(Asp160Asn)** |  |
| (NM_022124.5) | **c.1152C>A** | **p.(Ser384Arg)** |  |
|  | c.2263C>T | p.(His755Tyr) |  |
|  | c.2289+1G>A |  |  |
|  | **c.2330C>A** | **p.(Thr777Lys)** |  |
|  | **c.2566dupA** | **p.(Ile856Asnfs*7)** |  |
|  | **c.3220G>A** | **p.(Asp1074Asn)** |  |
|  | c.3367C>T | p.(Gln1123*) |  |
|  | **c.3388G>T** | **p.(Asp1130Tyr)** |  |
|  | **c.3821A>T** | **p.(Glu1274Val)** |  |
|  | c.4759_4766delACACGGCC | p.(Thr1587Cysfs*4) |  |
|  | **c.5292G>A** | **p.(Trp1764*)** |  |
|  | **c.5368+1G>A** |  |  |
|  | c.6049G>A | p.(Gly2017Ser) |  |
|  | c.6050-9G>A |  |  |
|  | **c.6396delC** | **p.(Ile2132Metfs*11)** |  |
|  | c.6442G>A | p.(Asp2148Asn) |  |
|  | **c.6829+2T>C** |  |  |
|  | **c.7482+1G>A** |  |  |
|  | **c.8432G>A** | **p.(Trp2811*)** |  |
|  | **c.9233_9234delTG** | **p.(Leu3078Argfs*41)** |  |
|  | **c.(145+1_146-1)_(429+1_430-1)del** | **del exons 4-6** |  |
|  | **c.(288+1_289-1)_(336+1_337-1)del** | **del exon 5** |  |
|  | **c.(336+1_337-1)_(1290+1_1291-1)dup** | **dup exons 6-12** |  |
|  | **c.(336+1_337-1)_(4617+1_4618-1)dup** | **dup exons 6-35** |  |
|  | **c.(429+1_430-1)_(832+1_833-1)dup** | **dup exons 7-9** |  |
| *PCDH15* | c.7C>T | p.(Arg3*) |  |
| (NG_009191.1) | **c.107-2A>G** |  |  |
| (NM_001142769.1) | c.748C>T | p.(Arg250*) |  |
|  | c.1021C>T | p.(Arg341*) |  |
|  | c.1103delT | p.(Leu368Trpfs*58) |  |
|  | **c.1986T>G** | **p.(Tyr662*)** |  |
|  | c.3753+1G>A |  |  |
|  | **c.4164dupC** | **p.(Leu1390Valfs*26)** |  |
|  | **c.5371T>C** | **p.(*1791Argext*5)** |  |
|  | **c.(?_-395)del** | **del promoter** |  |
|  | **c.(?_-395)_(91+1_92-1)del** | **del promoter + exons 1-2** |  |
|  | **c.(1113+1_1114-1)_(1320+1_1321-1)del** | **del exon 12** |  |
|  | **c.(1820+1_1821-1)_(2256+1_2257-1)del** | **del exon 17-20** |  |
| *USH1G* | c.113G>A | p.(Trp38*) |  |
| (NG_007882.1) | **c.387dupC** | **p.(Lys130Glnfs*5)** |  |
| (NM_173477.4) | **c.742C>T** | **p.(Gln248*)** |  |
|  | **c.800G>A** | **p.(Trp267*)** |  |
|  | **c.(?_-190)_(*1992_?)del** | **del exons 1-3** |  |
|  | **c.(1382+1_1383-1)_(*1992_?)del** | **del exon 3** |  |
| *USH2A* | c.100C>T | p.(Arg34*) |  |
| (NG_009497.1) | **c.232T>G** | **p.(Phe78Val)** |  |
| (NM_206933.2) | **c.269A>G** | **p.(Tyr90Cys)** |  |
|  | **c.486-2A>C** |  |  |
|  | **c.562T>A** | **p.(Tyr188Asn)** |  |
|  | **c.633G>A** | **p.(Trp211*)** |  |
|  | c.653T>A | p.(Val218Glu) |  |
|  | c.775_776delAG | p.(Ser259Phefs*63) |  |
|  | c.802G>A | p.(Gly268Arg) |  |
|  | **c.821G>A** | **p.(Arg274Gln)** |  |
|  | c.852_853delGA | p.(Glu284Aspfs*38) |  |
|  | c.854T>C | p.(Ile285Thr) |  |
|  | **c.859G>T** | **p.(Glu287*)** |  |
|  | c.908G>A | p.(Arg303His) |  |
|  | c.920_923dupGCCA | p.(His308Serfs*16) |  |
|  | **c.926C>T** | **p.(Pro309Leu)** |  |
|  | c.949C>A | p.(=,Tyr318Cysfs*17) |  |
|  | **c.990_991del** | **p.(Asn330Lysfs*8)** |  |
|  | c.1000C>T | p.(Arg334Trp) |  |
|  | **c.1018C>G** | **p.(His340Asp)** |  |
|  | c.1036A>C | p.(Asn346His) |  |
|  | c.1055C>T | p.(Thr352Ile) |  |
|  | **c.1144-2A>T** |  |  |
|  | c.1256G>T | p.(Cys419Phe) |  |
|  | c.1530C>A | p.(Glu511Lys) |  |
|  | **c.1546G>A** | **p.(Gly516Arg)** |  |
|  | c.1547G>T | p.(Gly516Val) |  |
|  | c.1558T>C | p.(Cys520Arg) |  |
|  | **c.1603_1606dupAGAT** | **p.(Cys536*)** |  |
|  | c.1841-2A>G |  |  |
|  | c.1876C>T | p.(Arg626*) |  |
|  | c.1978G>A | p.(Gly660Arg) |  |
|  | c.2071T>C | p.(Cys691Arg) |  |
|  | **c.2086T>A** | **p.(Cys696Ser)** |  |
|  | c.2149T>G | p.(Cys717Gly) |  |
|  | c.2209C>T | p.(Arg737*) |  |
|  | c.2276G>T | p.(Cys759Phe) |  |
|  | c.2296T>C | p.(Cys766Arg) |  |
|  | c.2299delG | p.(Glu767Serfs*21) |  |
|  | **c.2431A>T** | **p.(Lys811*)** |  |
|  | c.2610C>A | p.(Cys870*) |  |
|  | c.2755C>T | p.(Gln919*) |  |
|  | c.2802T>G | p.(Cys934Trp) |  |
|  | **c.2859C>A** | **p.(Cys953*)** |  |
|  | c.2993G>A | p.(Arg998Lys) |  |
|  | **c.3251G>A** | **p.(Trp1084*)** |  |
|  | c.3309C>A | p.(Tyr1103*) |  |
|  | **c.3317-1G>A** |  |  |
|  | c.3317-2A>G |  |  |
|  | c.3407G>A | p.(Ser1136Asn) |  |
|  | **c.3684T>A** | **p.(Cys1228*)** |  |
|  | **c.3722_3729delinsTCA** | **p.(Ala1241Valfs*8)** |  |
|  | c.3737dupT | p.(Ser1247Lysfs*4) |  |
|  | **c.3890_3891delTT** | **p.(Phe1297Serfs*17)** |  |
|  | **c.3950T>A** | **p.(Leu1317*)** |  |
|  | **c.4049delC** | **p.(Ser1350Leufs*16)** |  |
|  | c.4222C>T | p.(Gln1408*) |  |
|  | **c.4327A>C** | **p.(Thr1443Pro)** |  |
|  | [c.4474G>T](https://grenada.lumc.nl/LOVD2/Usher_montpellier/variants.php?select_db=USH2A&action=search_all&search_Variant%2FDNA=c.4474G%3ET) | p.Glu1492* |  |
|  | c.4645C>T | p.(Arg1549*) |  |
|  | **c.4668delG** | **p.(Ile1557Leufs*18)** |  |
|  | c.4714delC | p.(Leu1572Phefs*3) |  |
|  | **c.4717C>T** | **p.(Gln1573*)** |  |
|  | c.4758+1G>A |  |  |
|  | **c.4933G>T** | **p.(Gly1645*)** |  |
|  | c.4957C>T | p.(Arg1653*) |  |
|  | **c.5001dupA** | **p.(Gly1668Argfs*30)** |  |
|  | **c.5129_5130insGAGC** | **p.(Ala1711Serfs*6)** |  |
|  | c.5329C>T | p.(Arg1777Trp) |  |
|  | **c.5447T>G** | **p.(Val1816Gly)** |  |
|  | **c.5522G>A** | **p.(Gly1841Glu)** |  |
|  | c.5528C>T | p.(Pro1843Leu) |  |
|  | c.5588T>C | p.(Met1863Thr) |  |
|  | **c.5698T>G** | **p.(Cys1900Gly)** |  |
|  | c.5776+1G>A |  |  |
|  | **c.5776+2T>C** |  |  |
|  | **c.5777-2A>G** |  |  |
|  | **c.6225G>T** | **p.(Trp2075Cys)** |  |
|  | c.6383G>A | p.(Cys2128Tyr) |  |
|  | **c.6657+1G>C** |  |  |
|  | **c.6721C>A** | **p.(Pro2241Thr)** |  |
|  | **c.6722C>A** | **p.(Pro2241His)** |  |
|  | **c.6724G>T** | **p.(Glu2242*)** |  |
|  | c.6730G>A | p.(Val2244Met) |  |
|  | c.6805+2T>C |  |  |
|  | c.6862G>T | p.(Glu2288*) |  |
|  | c.6926G>T | p.(Cys2309Phe) |  |
|  | c.6956delT | p.(Leu2319Argfs*7) |  |
|  | c.7139_7140delTT | p.(Leu2380Profs*37) |  |
|  | **c.7452-1G>A** |  |  |
|  | c.7524delT | p.(Arg2509Glyfs*19) |  |
|  | c.7595-2144A>G |  |  |
|  | c.7595-3C>G |  |  |
|  | **c.7697delA** | **p.(Tyr2566Leufs*11)** |  |
|  | c.7915T>C | p.(Ser2639Pro) |  |
|  | **c.7950dupC** | **p.(Asn2651Glnfs*10)** |  |
|  | **c.8099T>A** | **p.(Val2700Glu)** |  |
|  | **c.8143delG** | **p.(Val2715*)** |  |
|  | **c.8522G>A** | **p.(Trp2841*)** |  |
|  | c.8557A>T | p.(Arg2853*) |  |
|  | **c.8698T>A** | **p.(Tyr2900Asn)** |  |
|  | c.8740C>T | p.(Arg2914*) |  |
|  | c.8755A>C | p.(Thr2919Pro) |  |
|  | c.8834G>A | p.(Trp2945*) |  |
|  | **c.8906C>G** | **p.(Ser2969*)** |  |
|  | **c.9014G>C** | **p.(Ser3005Thr)** |  |
|  | **c.9056-1_9056delinsTC** |  |  |
|  | **c.9258+1G>A** |  |  |
|  | c.9270C>A | p.(Cys3090*) |  |
|  | **c.9307A>G** | **p.(Ile3103Val)** |  |
|  | c.9345_9346delAC | p.(Pro3116Hisfs*13) |  |
|  | **c.9355C>T** | **p.(Arg3119Cys)** |  |
|  | c.9371+1G>T |  |  |
|  | c.9424G>T | p.(Gly3142*) |  |
|  | **c.9791delG** | **p.(Gly3264Valfs*37)** |  |
|  | c.9799T>G | p.(Cys3267Arg) |  |
|  | c.9811delA | p.(Met3271Cysfs*30) |  |
|  | c.9815C>T | p.(Pro3272Leu) |  |
|  | c.9862G>C | p.(Asp3288His) |  |
|  | c.9874C>T | p.(Gln3292*) |  |
|  | **c.10183G>T** | **p.(Glu3395*)** |  |
|  | **c.10481C>G** | **p.(Thr3494Arg)** |  |
|  | c.10561T>C | p.(Trp3521Arg) |  |
|  | c.10612C>T | p.(Arg3538*) |  |
|  | c.10712C>T | p.(Thr3571Met) |  |
|  | c.10759C>T | p.(Gln3587*) |  |
|  | c.10817T>C | p.(Leu3606Pro) |  |
|  | c.10852G>A | p.(Gly3618Ser) |  |
|  | c.10939G>A | p.(Gly3647Ser) |  |
|  | c.10979delC | p.(Ala3660fsVal*14) |  |
|  | **c.11027C>T** | **p.(Thr3676Ile)** |  |
|  | c.11005G>A | p.(Trp3702*) |  |
|  | **c.11145T>G** | **p.(Tyr3715*)** |  |
|  | c.11156G>A | p.(Arg3719His) |  |
|  | **c.11389+3A>G** |  |  |
|  | c.11548+2T>G |  |  |
|  | c.11549-1G>A |  |  |
|  | c.11566delA | p.(Ser3856Valfs*28) |  |
|  | **c.11594C>A** | **p.(Ala3865Glu)** |  |
|  | **c.11819A>G** | **p.(Tyr3940Cys)** |  |
|  | c.11831C>A | p.(Ala3944asp) |  |
|  | c.11864G>A | p.(Trp3955*) |  |
|  | c.11875_11876delCA | p.(Gln3959Asnfs*53) |  |
|  | c.12067-2A>G |  |  |
|  | c.12093delC | p.(Tyr4031*) |  |
|  | **c.12094G>A** | **p.(Gly4032Arg)** |  |
|  | **c.12175dupA** | **p.(Ile4059Asnfs*40)** |  |
|  | c.12234_12235delGA | p.(Asn4079Trpfs*19) |  |
|  | **c.12283G>A** | **p.(Gly4095Ser)** |  |
|  | c.12284G>A | p.(Gly4095Asp) |  |
|  | **c.12302_12304delACA** | **p.(Asn4101del)** |  |
|  | c.12343C>T | p.(Arg4115Cys) |  |
|  | c.12381_12382delCT | p.(Tyr4128Hisfs*24) |  |
|  | **c.12419G>A** | **p.(Cys4140Phe)** |  |
|  | c.12574C>T | p.(Arg4192Cys) |  |
|  | **c.12695C>T** | **p.(Pro4232Leu)** |  |
|  | c.12703C>T | p.(Gln4235*) |  |
|  | c.12485C>T | p.(Leu4282Pro) |  |
|  | **c.12893A>G** | **p.(Tyr4298Cys)** |  |
|  | **c.12900dupT** | **p.(Gln4301Serfs*4)** |  |
|  | **c.12943A>C** | **p.(Thr4315Pro)** |  |
|  | c.12954C>A | p.(Tyr4318*) |  |
|  | **c.12987T>G** | **p.(Tyr4329*)** |  |
|  | c.13010C>T | p.(Thr4337Met) |  |
|  | **c.13018G>C** | **p.(Gly4340Arg)** |  |
|  | **c.13231_13238dup** | **p.(Tyr4414Cysfs*12)** |  |
|  | **c.13268C>A** | **p.(Ala4423Asp)** |  |
|  | c.13274C>T | p.(Thr4425Met) |  |
|  | c.13316C>T | p.(Thr4439Ile) |  |
|  | c.13374delA | p.(Glu4458Aspfs*3) |  |
|  | c.13700delT | p.(Leu4567Profs*16) |  |
|  | c.13811+2T>G |  |  |
|  | c.13822C>T | p.(Arg4608*) |  |
|  | **c.13903C>T** | **p.(Gln4635*)** |  |
|  | **c.14026dupC** | **p.(Gln4676Profs*7)** |  |
|  | **c.14091delT** | **p.(Phe4697Leufs*2)** |  |
|  | c.14131C>T | p.(Gln4711*) |  |
|  | c.14174G>A | p.(Trp4725*) |  |
|  | **c.14204C>G** | **p.(Pro4735Arg)** |  |
|  | c.14225_14232dupCGTTCCAT | p.(Val4745Argfs*4) |  |
|  | **c.14248C>T** | **p.(Gln4750*)** |  |
|  | c.14287G>A | p.(Gly4763Arg) |  |
|  | c.14426C>T | p.(Thr4809Ile) |  |
|  | **c.14450G>A** | **p.(Gly4817Glu)** |  |
|  | c.14519T>C | p.(Leu4840Pro) |  |
|  | **c.14570G>C** | **p.(Gly4857Ala)** |  |
|  | **c.14791+2T>C** |  |  |
|  | **c.14791+4A>G** |  |  |
|  | c.14791+5G>T |  |  |
|  | c.14803C>T | p.(Arg4935*) |  |
|  | **c.14885dupA** | **p.(Glu4963Glyfs*38)** |  |
|  | c.14977_14978delTT | p.(Phe4993Profs*7) |  |
|  | c.14996C>T | p.(Thr4999Ile) |  |
|  | **c.15017C>T** | **p.(Thr5006Met)** |  |
|  | c.15089C>A | p.(Ser5030*) |  |
|  | c.15364T>C | p.(Cys5122Arg) |  |
|  | **c.15380delC** | **p.(Pro5127Argfs*8)** |  |
|  | c.15428G>A | p.(Arg5143His) |  |
|  | **c.15496A>G** | **p.(Ile5166Val)** |  |
|  | **c.(651+1_652-1)_(784+1_785-1)del** | **del exon 4** |  |
|  | **c.(784+1_785-1)_(848+1_849-1)del** | **del exon 5** |  |
|  | c.785-6636_1840+208del | del exons 5-10 |  |
|  | **c.(1550+1_1551-1)_(4627+1_4628-1)del** | **del exons 9-21** |  |
|  | **c.(1644+1_1645-1)_(4627+1_4628-1)del** | **del exons 10-21** |  |
|  | **c.(2993+1_2994-1)_(3157+1_3158-1)del** | **del exon 15** |  |
|  | **c.(2993+1_2994-1)_(13811+1_13812-1)dup** | **dup exons 15-63** |  |
|  | **c.(3157+1_3158-1)_(4627+1_4628-1)del** | **del exons 16-21** |  |
|  | **c.(3316+1_3317-1)_(4627+1_4628-1)del** | **del exons 17-21** |  |
|  | c.(4251+1_4252-1)_(4396+1_4397-1)del | del exon 20 |  |
|  | c.4627+25435_4987+660del | del exons 22-24 |  |
|  | **c.(4885+1_4886-1)_(5572+1_5573-1)del** | **del exons 24-27** |  |
|  | **c.(6163+1_6164-1)_(6325+1_6326-1)del** | **del exon 32** |  |
|  | **c.(6658+1_6659-1)_(6805+1_6806-1)del** | **del exon 35** |  |
|  | c.9259-2402_9371+1537del | del exon 47 |  |
|  | **c.(11548+1_11549-1)_(11711+1_11712-1)del** | **del exon 60** |  |
| *ADGRV1* | **c.1477C>T** | **p.(Arg493*)** |  |
| (NG_007083.1) | **c.1509+3A>G** |  |  |
| (NM_032119.3) | **c.1608C>G** | **p.(Tyr536*)** |  |
|  | **c.1701delC** | **p.(Leu568Cysfs*8)** |  |
|  | c.2258_2270delAAGTGCTGAAATC | p.(Gln753Leufs*8) |  |
|  | c.2864C>A | p.(Ser955*) |  |
|  | **c.4072T>C** | **p.(Ser1358Pro)** |  |
|  | c.5357_5358delAA | p.(Lys1786Ilefs*8) |  |
|  | **c.6093delA** | **p.(Ala2032Argfs*27)** |  |
|  | **c.6133G>T** | **p.(Gly2045*)** |  |
|  | **c.6984_6985delTG** | **p.(Gly2329Argfs*17)** |  |
|  | **c.8005delG** | **p.(Glu2669Lysfs*4)** |  |
|  | **c.9184+3A>G** |  |  |
|  | **c.9877C>T** | **p.(Arg3293*)** |  |
|  | **c.9906+1G>A** |  |  |
|  | **c.10054-1G>T** |  |  |
|  | c.10458G>A | p.(Trp3486*) |  |
|  | **c.10468C>T** | **p.(Gln3490*)** |  |
|  | **c.10527dupC** | **p.(Phe3510Leufs*28)** |  |
|  | **c.10768A>T** | **p.(Ser3590Cys)** |  |
|  | c.11974G>A | p.(Asp3992Asn) |  |
|  | **c.12143C>A** | **p.(Ser4048*)** |  |
|  | c.12555_12556delGG | p.(Glu4186Glyfs*17) |  |
|  | c.13320dupC | p.(Ser4441Leufs*9) |  |
|  | **c.14153delT** | **p.(Leu4718Cysfs*9)** |  |
|  | **c.15981delC** | **p.(Phe5328Serfs*41)** |  |
|  | **c.16876C>T** | **p.(Gln5626*)** |  |
|  | c.16940delT | p.(Val5647Glyfs*7) |  |
|  | c.17668_17669delAT | p.(Met5890Valfs*10) |  |
|  | **c.17909G>C** | **p.(Cys5970Ser)** |  |
|  | **c.18025C>T** | **p.(Arg6009*)** |  |
|  | **c.18884_18887del** | **p.(Glu6295Alafs*31)** |  |
|  | **c.(3022+1_3023-1)_(16611+1_16612-1)dup** | **del exons 17-77** |  |
|  | **c.(4378+1_4379-1)_(16196+1_16197-1)del** | **del exons 21-75** |  |
|  | **c.6951+1_6952-1)_(7133+1_7134-1)del** | **del exon 32** |  |
| *PDZD7*  (NG_028030.1)  (NM_001195263.1) | **c.2806C>T** | **p.(Arg936*)** |  |
| *CLRN1* | c.144T>G | p.(Asn48Lys) |  |
| (NG_009168.1) | c.149_152delCAGGinsTGTCCAAT | p.(Ser50Leufs*12) |  |
| (NM_174878.2) | **c.176delG** | **p.(Gly59Valfs*13)** |  |
|  | **c.227dupT** | **p.(Leu76Phefs*54)** |  |
|  | **c.230G>A** | **p.(Gly77Glu)** |  |
|  | **c.230dupG** | **p.(Ala78Serfs*52)** |  |
|  | **c.434-2A>T** |  |  |
|  | c.368C>A | p.(Ala123Asp) |  |
|  | c.504dupT | p.(Ile168Asnfs*5) |  |
| (NM_052995.2) | **c.300T>G** | **p.(Tyr100*)** |  |
|  |  |  |  |
|  |  |  |  |

Novel mutations are indicated in bold
